# Malnutrition exacerbating neuropsychiatric symptoms on the Alzheimer's continuum is relevant to the cAMP signaling pathway: Human and mouse studies

**DOI:** 10.1002/alz.14506

**Published:** 2025-01-27

**Authors:** Jiwei Jiang, Tianlin Jiang, Xiaohong Wang, Min Zhao, Hanping Shi, Huiying Zhang, Wenyi Li, Shirui Jiang, Xiaoli Zhang, Jiawei Zhou, Qiwei Ren, Linlin Wang, Shiyi Yang, Zeshan Yao, Yaou Liu, Jun Xu

**Affiliations:** ^1^ Beijing Tiantan Hospital, Capital Medical University Beijing China; ^2^ China National Clinical Research Center for Neurological Diseases Beijing China; ^3^ Institute of Translational Medicine Medical College Yangzhou University Yangzhou China; ^4^ Jiangsu Key Laboratory of Experimental & Translational Non‐coding RNA Research Yangzhou University Yangzhou China; ^5^ Beijing Shijitan Hospital, Capital Medical University Beijing China; ^6^ Beijing International Science and Technology Cooperation Base for Cancer Metabolism and Nutrition Beijing China; ^7^ Beijing Institute of Collaborative Innovation Beijing China

**Keywords:** Alzheimer's disease, cyclic adenosine monophosphate, malnutrition, midbrain, neuropsychiatric symptoms, neurotransmitter

## Abstract

**INTRODUCTION:**

Malnutrition correlates with neuropsychiatric symptoms (NPSs) in Alzheimer's disease (AD); however, the potential mechanism underlying this association remains unclear.

**METHODS:**

Baseline and longitudinal associations of nutritional status with NPSs were analyzed in 374 patients on the AD continuum and 61 healthy controls. Serum biomarkers, behavioral tests, cerebral neurotransmitters, and differentially gene expression were evaluated in standard and malnourished diet–fed transgenic APPswe/PSEN1dE9 (APP/PS1) mice.

**RESULTS:**

Poor nutritional status and increased cerebral blood flow in the midbrain and striatum were associated with severe general NPSs and subtypes, especially depression, anxiety, and apathy. APP/PS1 mice fed a malnourished diet showed poor nutritional status, depression‐ and anxiety‐like behaviors, altered neurotransmitter levels, and downregulated *c‐Fos* expression in the midbrain and striatum; these were associated with suppressed cyclic adenosine monophosphate (cAMP) signaling pathway.

**DISCUSSION:**

Malnutrition exacerbating NPSs is relevant to suppressed cAMP pathway in the midbrain and striatum, suggesting the potential for targeted nutritional interventions to mitigate NPSs in the AD continuum.

**Highlights:**

- Poor nutritional status linked to general and specific neuropsychiatric symptom (NPS) deterioration.
- Malnutrition affects NPSs, usually involving the midbrain and striatum.
- Malnourished diet induces depression‐ and anxiety‐like behaviors in APP/PS1 mice.
- Malnutrition exacerbates NPSs associated with cAMP signaling pathway in the midbrain and striatum.

## 1 BACKGROUND

Dietary nutrition is an important factor that directly or indirectly influences Alzheimer's disease (AD) through complex interactions among behavioral, genetic, systemic, and cerebral factors.^1^, ^2^ A longitudinal study found that weight loss could predict increased tau and phospho‐tau levels, which occurred downstream of amyloid beta (Aβ) accumulation in preclinical AD, paralleling cognitive decline.^3^ Another longitudinal study found that poor nutritional status and unhealthy dietary patterns were associated with a high risk of clinical progression in 551 patients on the AD continuum.^4^ However, for the AD continuum, accumulating knowledge has focused on the association between nutrition and cognition than that on neuropsychiatric symptoms (NPSs).^1^, ^5^ NPSs comprise an important and prevalent group of highly heterogeneous “non‐cognitive” symptoms, which are generally associated with faster disease progression and heavier caregiver burden.^6^, ^7^ Various biopsychosocial factors contribute to the onset and development of NPSs, including neurobiologically related disease factors, the disease itself, caregiver factors, environmental triggers, and their interactions, which also limits NPS management.^8^, ^9^ Accordingly, the complex interplay of multiple biological pathways and multifaceted effects of nutrition may provide new insights into elucidating and managing NPSs. To achieve this goal, a three‐step “up‐bottom” multi‐pronged approach is required: first, identifying nutrition‐related risk factors that frequently co‐occur with NPS in patients with AD.^10^ Second, establishing a prospective human cohort to reveal the effect of the multi‐omics measurements of malnutrition on the onset and development of NPSs in patients on the AD continuum. Finally, developing an animal model to reveal the shared mechanisms between these nutritional risk factors and NPSs as potential interventional targets.^11^

In our previous study, we developed and validated a nomogram, encompassing a set of nutrition‐related genetic/clinical/radiological risk factors, including cholesteryl ester transfer protein (*CETP*) rs1800775*A polymorphism, decreased nutritional status, increased caregiver burden, and decreased brainstem volume, to predict NPS occurrence in patients with AD, suggesting the potential multidimensional role of nutrition in the presence of NPSs.^12^ Similarly, recent studies have revealed that malnutrition or risk of malnutrition was associated with individual NPSs in older women with mild cognitive impairment (MCI) and early‐stage AD.^13^, ^14^ Another longitudinal study demonstrated that poorer nutritional status was associated with more severe general NPSs and its sub‐symptoms, including psychosis, depression, and apathy, in patients with dementia.^15^ This evidence preliminarily demonstrates an association between malnutrition and NPS development; however, the included population was highly heterogeneous (including only women with AD or patients with various types of dementia). Moreover, nutritional assessment in previous studies has been limited to clinical scales, and the evaluation of the characteristics of malnutrition using serological biomarkers and multimodal neuroimaging remains unexplored.^1^, ^16^ Notably, the potential mechanism underlying the association between malnutrition and NPSs remains unknown. Our preliminary research has revealed that brainstem atrophy and abnormal putamen perfusion regulates the association between malnutrition and NPSs, suggesting that they may share a common neural regulatory basis.^12^, ^17^ Thus, in the present study, we aimed to investigate the effect of the multi‐omics measurements of malnutrition on NPS onset and development in patients on the AD continuum based on a prospective human cohort study. Furthermore, we aimed to reveal the underlying pathway in the brain regions of interest (ROIs) relevant to the association between malnutrition and NPSs using transgenic APPswe/PSEN1dE9 (APP/PS1) mice. These findings are expected to elucidate the association between malnutrition and NPSs and the mechanisms underlying their association on the AD continuum to provide novel insights into potential interventional targets for NPSs.

## 2 METHODS

### 2.1 Human cohort study

**FIGURE 1 alz14506-fig-0001:**
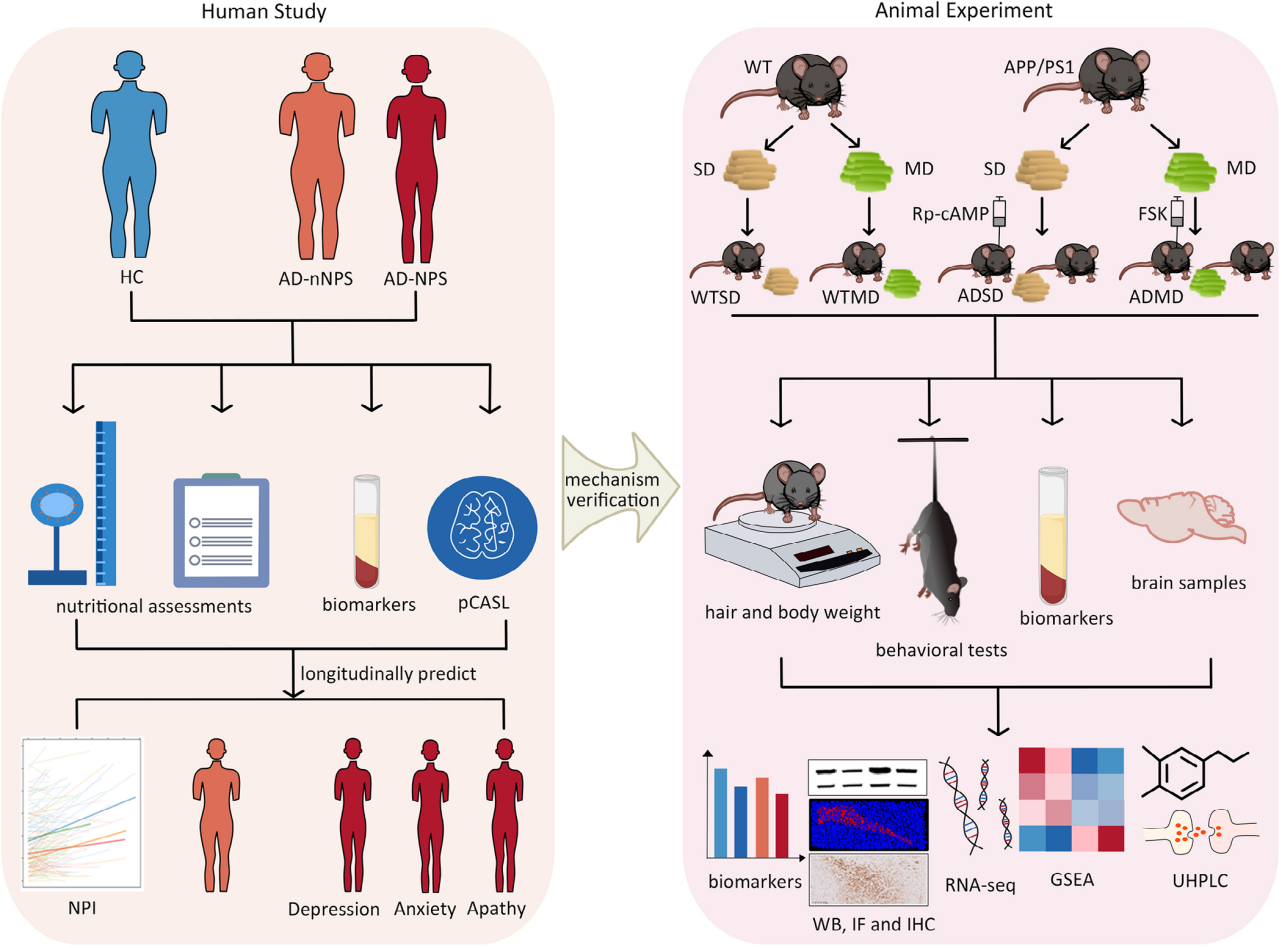
Detailed schematic representation of the human cohort study and animal experimental design. AD, Alzheimer's disease; AD‐nNPS, patients without NPSs on the AD continuum; AD‐NPS: patients with NPSs on the AD continuum; APP/PS1, transgenic APPswe/PSEN1dE9; FSK, forskolin; GSEA, Gene Set Enrichment Analysis; HC, healthy control; IF, immunofluorescence; IHC, immunohistochemistry; MD, malnourished diet; NPI, Neuropsychiatric Inventory; NPS, neuropsychiatric symptom; pCASL, pseudo‐continuous arterial spin labeling; RNA‐seq, ribonucleic acid sequencing; Rp‐cAMP, a specific inhibitor of cyclic adenosine monophosphate signaling pathway; SD, standard diet; UHPLC, ultra‐high‐performance liquid chromatography; WB, western blot; WT, wild type.

Figure 1 schematically represents the human study and animal experimental design. The human study included 374 patients on the AD continuum and 61 healthy controls (HCs) from the Chinese Imaging, Biomarkers, and Lifestyle (CIBL) study between May 1, 2021, and May 1, 2023. The CIBL study, as described previously,^18^, ^19^ was approved by the ethics committee of Capital Medical University, Beijing Tiantan Hospital (approval number: KY‐2021‐028‐01), and registered at chictr.org.cn (ChiCTR2100049131). All participants or their legally authorized caregivers (if applicable) were informed of the purpose of this study and provided written informed consent. The detailed inclusion and exclusion criteria are specified in Supplementary Material S01.

Trained research coordinators interviewed patients on the AD continuum personally at ≈6‐month intervals. The assessment parameters were identical to those at baseline. In the absence of face‐to‐face follow‐ups, information was obtained by telephone.

### 2.2 Clinical data and neuropsychological assessment

Baseline data regarding demographics and clinical characteristics (including age, sex, body mass index [BMI], waist‐to‐hip ratio, years of education, marital status, socioeconomic status [SES], and duration from disease diagnosis to initial enrollment), medical history (hypertension, diabetes mellitus, cerebrovascular disease, coronary heart disease, and dyslipidemia), smoking history, and alcohol consumption, were collected at admission. The individual's comprehensive SES was measured by adding the scores of education level, income level, and professional status, as described previously for the Chinese population.^20^ The detailed calculations for the SES scores are described in Supplementary Material S02. Smoking history includes current active smoking history, smoking duration, daily smoking volume, and passive smoking history. Laboratory parameters related to nutritional status included complete blood counts, apolipoprotein E ε4 allele (*APOE* ε4) status, and serum levels of albumin, glucose, triglyceride, total cholesterol (TC), blood urea nitrogen (BUN), vitamin B12, folic acid, vitamin D3, and vitamin D. As described previously, these serum biomarkers serve as objective nutritional tools, including the controlling nutritional status score, prognostic nutritional index (PNI), and geriatric nutritional risk index (GNRI), that are used to evaluate malnutrition risk.^21^ The detailed calculations for these three objective nutritional tools are described in Supplementary Material S03.

**RESEARCH IN CONTEXT**

1. **Systematic review**: A systematic review was conducted in PubMed and Web of Science until April 2024. No studies so far have elucidated the longitudinal impact and underlying biological mechanisms of malnutrition on neuropsychiatric symptoms (NPSs) in patients with Alzheimer's disease (AD) and APP/PS1 mice.
2. **Interpretation**: The study identified a novel relationship between malnutrition and NPSs in both patients on the AD continuum and APP/PS1 mice. A malnourished diet induced depression‐ and anxiety‐like behaviors in APP/PS1 mice by affecting the balance of neurotransmitter levels in the midbrain and striatum via the cAMP/c‐Fos signaling pathway. It provides novel insights for early targeted nutritional interventions on NPSs.
3. **Future directions**: Future research, including prospective studies and clinical trials, should focus on identifying the best strategies to prevent and treat NPSs via targeting the cAMP signaling pathway, especially in patients with early malnutrition on the AD continuum.

Neuropsychological and nutritional tests, including the Neuropsychiatric Inventory (NPI), Hamilton Depression Scale (HAMD), 14‐item Hamilton Anxiety Scale (HAMA), Mini‐Mental State Examination (MMSE), Beijing version of the Montreal Cognitive Assessment (MoCA), Caregiver Burden Inventory (CBI), Mini‐Nutritional Assessment (MNA), and dietary diversity score were administered by a trained neurologist and neuropsychologist. The detailed evaluation content and standards for each scale are provided in Supplementary Material S04.[Fig alz14506-fig-0001]

### 2.3 Multimodal neuroimaging measures

A 3.0 T magnetic resonance (MR) scanner (SIGNA Premier; GE Healthcare, Milwaukee, WI, USA) with a 48‐channel head coil was used for image acquisition, including high‐resolution three‐dimensional (3D) T1‐weighted imaging and seven‐delay pseudo‐continuous arterial spin labeling. All participants were requested to refrain from consuming caffeine and alcohol for 8 h prior to undergoing MR imaging (MRI). The detailed scan parameters and image processing are shown in Supplementary Material S05. The ROIs in the brain are involved in both homeostatic regulation and cognitive control of eating.^22^, ^23^, ^24^ Based on our previous data on the association of brainstem atrophy, abnormal corrected cerebral blood flow (CBF) of the putamen, and ventral tegmental area (VTA) with NPSs,^12^, ^17^ in the present study, we used the corrected CBF data of 10 ROIs using the third edition of the automated anatomical labeling atlas (AAL3),^25^ including the bilateral hypothalamus, amygdala, insula, putamen, and VTA.

### 2.4 Animal experiments

Double transgenic APP/PS1 mice with a B6C3‐Tg background and wild‐type (WT) littermates (4 months old, weighing 22–30 g at baseline) were purchased from Hangzhou Ziyuan Laboratory Animal Technology Co., Ltd. (Yangzhou, China). The animals were maintained at the Yangzhou University Medical School's Institutional Specific‐Pathogen‐Free Animal Laboratory. They were housed in microisolator cages at a temperature of 23°C and humidity of 50 ± 15% on a 12:12 light–dark cycle with ad libitum access to water and food. This study was performed in accordance with the regulations of the Animal Experiment Ethics Committee of Yangzhou University (YXYLL‐2022‐71).

The APP/PS1 mice were randomly divided into two groups (*n* = 8/group, four male and four female) and fed an isocaloric standard or malnourished diet for ≈8 weeks, whereas the WT mice were grouped and intervened as described earlier. All diets were purchased from Xiao Shu You Tai Biotechnology. Co., Ltd. (Beijing, China). The fat and protein contents differed between the standard diet (SD; D09051102Ni) and the malnourished diet (MD; D10062201; https://researchdiets.com/).^26^ Detailed compositions of the diets are provided in Supplementary Material S06. The compositions of both diets, tested by the PONY Testing International Group (Beijing, China), were consistent with those of the formula ingredients. The four groups were as follows: (1) WTSD: WT mice fed a SD; (2) WTMD: WT mice fed a MD; (3) ADSD: APP/PS1 mice fed a SD; and (4) ADMD: APP/PS1 mice fed a MD.

The body weights and fur of the mice were analyzed at 0, 2, 4, 6, and 8 weeks. The baseline blood biomarkers (hemoglobin, albumin, glucose, TC, triglyceride, BUN, vitamin B12, and vitamin D3 levels) were obtained from the ophthalmic vein of all non‐fasted and fasted (overnight) mice and immediately analyzed before dietary intervention. The same biomarkers were analyzed following behavioral testing after an 8‐week dietary intervention. Routine blood was measured using the BC‐2800 Vet Animal Auto Hematology Analyzer (MINDRAY, Shenzhen, China), serum biochemical markers were analyzed using a Beckman Coulter AU480 Automated Chemistry Analyzer (Beckman Coulter, CA, USA), and plasma vitamins were analyzed using ultra‐high performance liquid chromatography‐tandem triple quadrupole mass spectrometry.

### 2.5 Behavioral testing

After the 8‐week dietary intervention, behavioral tests—including the open field test (OFT), tail suspension test (TST), forced swim test (FST), and sucrose‐preference test (SPT)—were performed to assess the effect of the malnourished diet on NPS‐like behavior in APP/PS1 mice. The Morris water‐maze or Y‐maze test was used to evaluate cognition (spatial learning and memory). A detailed description of each behavioral test has been presented in Supplementary Material S07.

Behavioral testing was performed during the night of the diurnal cycle (08:00 p.m. to 12:00 p.m.) under conditions of dim light (10 lx) and low noise. All behavioral procedures were performed by the same two experienced researchers, who were blinded to the type of intervention. To minimize the stress associated with the procedures, all mice were acclimatized to the handling and soundproof testing environment for at least 1 h before the start of each trial. The device was cleaned with 75% ethanol after each test to remove any residual odor. The behavioral apparatus and digital video camera used to record the experimental data were purchased from Shanghai Xinruan Co., Ltd. (Shanghai, China).

### 2.6 Structural MRI of mouse brains

Three days after behavioral testing, the mice from the four groups were anesthetized using 1.25% 2,2,2‐tribromoethanol (*n* = 2 mice, 0.02 mL/g; administered intraperitoneally). All solutions were placed in the dark. A 7.0 T BioSpec70/20 USR Analyzing and Imaging system (Bruker, Co. Ltd., Germany) was used for T2‐weighted TurboRARE sequence imaging. The parameters were as follows: slice thickness = 0.5 mm, repetition time (TR) = 3500.0 ms, echo time (TE) = 20.0 ms, number of excitations = 2.0, field of view = 15 × 15 mm^2^, flip angle = 90°, matrix size = 120 × 120, scan duration = 3.5 min, and slice number = 22–24. All operators were blinded to the group allocation. The ROI volumes were segmented manually using 3D Slicer, a freely available online tool.^27^

### 2.7 Western blot, immunofluorescence, and immunohistochemistry

Five mice from each group were deeply anesthetized and transcranially perfused with heparin‐containing saline, followed by 4% paraformaldehyde solution. The brains were quickly removed and the brains from three mice per group divided into two hemispheres, fixed with paraformaldehyde, and preserved at −20°C for a maximum of 3 months. Proteins in the midbrain or striatum from one of the hemispheres were extracted for western blot (WB) to investigate the expression levels of four neurotransmitters. The other hemispheres post‐fixed in 4% paraformaldehyde overnight at 4 °C were used for immunofluorescence (IF) to investigate the quantification and distribution of neurotransmitters in the midbrain and striatum. The other brains from two mice in each group were embedded in paraffin, serially sectioned (thickness, 4 µm), and mounted on silane‐covered slides for immunohistochemistry (IHC). The detailed procedures for WB, IHC, and IF, based on previously described protocols, are shown in Supplementary Material S08,^28^, ^29^ and the details of primary antibodies used in these experiments are shown in Table S1.

### 2.8 Quasi‐targeted metabolomic analysis

Quasi‐targeted metabolomics using ultra‐high‐performance liquid chromatography‐tandem mass spectrometry (QTrap 6500 plus, AB Sciex Co., Ltd., USA) was used to analyze 39 neurotransmitters such as acetylcholine, 5‐hydroxyindoleacetic acid, serotonin (5‐HT), dopamine, γ‐aminobutyric acid (GABA), and spermidine, and their metabolites.^30^, ^31^ Brain tissues (midbrain or striatum) from four mice in each group were extracted and given to Biotree Biotech Co. Ltd. (Shanghai, China) for analysis; the detailed steps are shown in Supplementary Material S09. Differences in the midbrain or striatum neurotransmitters among the four groups, particularly between ADMD and ADSD, and the metabolic pathways enriched by these differential neurotransmitters were further analyzed using the Kyoto Encyclopedia of Genes and Genomes (KEGG) database.

### 2.9 Transcriptomic analysis of brain tissues

To analyze the effect of the malnourished diet on the specific brain regional total transcriptome, whole‐transcriptome sequencing of the midbrain (200 mg) or corpus striatum (20 mg) was performed to identify dysregulated microRNAs (miRNAs) among the four groups. Total RNA was extracted from the brain using TRIzol Reagent (Thermo Fisher Scientific, USA). RNA sequencing was performed at Wuhan MetWare Biotechnology Co., Ltd. (www.metware.cn) using an Illumina Genome Analyzer II system. Functional annotation analysis of differential genes was performed using Annotation, Visualization, and Integrated Discovery (https://david.ncifcrf.gov/home.jsp). The sequencing and analysis steps are detailed in Supplementary Material S10. Differentially expressed genes (DEGs) were analyzed using the DEseq package in R, and their enriched biological pathways were further analyzed using the Gene Ontology (GO) and KEGG databases. The T‐cell receptor repertoire of the samples was analyzed using MiXCR software (available at http://mixcr.milaboratory.com/ and https://github.com/milaboratory/mixcr/).

### 2.10 In vivo verification of the cyclic adenosine monophosphate signaling pathway influenced by malnutrition and associated with worsened NPSs

After 8‐week interventions, three mice from each group were transcranially perfused with heparin‐containing saline, and then with 4% paraformaldehyde solution. The brains were fixed with paraformaldehyde and preserved at −20°C for a maximum of 3 months. The cyclic adenosine monophosphate (cAMP) content and the expression levels of the key related proteins, including phosphorylated protein kinase A (p‐PKA, an activated form of PKA) and phospho‐cAMP response element binding (p‐CREB, an activated form of CREB) in the midbrain and striatum were determined using WB.

To further explore the underlying mechanism of the cAMP signaling pathway influenced by the malnutrition and associated with worsened NPSs, the activator of cAMP forskolin (FSK) or the specific inhibitor of cAMP signaling pathway (Rp‐cAMP) were administered to the APP/PS1 mice by intracerebroventricular injection (i.c.v.) during the first three consecutive days of dietary intervention. The APP/PS1 mice were randomly divided into six groups (*n* = 10/group): (1) ADSD control: APP/PS1 mice fed a SD; (2) ADSD+Rp‐cAMP: APP/PS1 mice with administration by i.c.v. Rp‐cAMP fed a SD; (3) ADSD+Sham: APP/PS1 mice with administration by i.c.v. saline fed a SD; (4) ADMD control: APP/PS1 mice fed a MD; (5) ADMD+FSK: APP/PS1 mice with administration by i.c.v. FSK fed a MD; and (6) ADMD+Sham: APP/PS1 mice with administration by i.c.v. saline fed a MD. After 2 months, the body weight, behavior, and serum nutritional biomarkers for each group were collected. The cAMP and p‐PKA expression levels in both the midbrain and striatum were determined using WB (*n* = 3/group) after behavioral testing. The detailed procedures were the same as those in Supplementary Material S08, and the details of the primary antibodies used in these experiments are shown in Table S1.

### 2.11 Statistical analyses

Categorical variables are expressed as numbers (proportions) and were assessed using the χ^2^ test, whereas continuous variables with a normal distribution are expressed as mean (SD) and were evaluated through univariate analysis of variance (ANOVA) or Student's *t*‐test. Continuous variables for skewed data are presented as medians with interquartile ranges (IQRs) and evaluated using the Kruskal–Wallis or Mann–Whitney *U* tests. Multivariate logistic regression models were used to determine the clinical, serological, and multimodal neuroimaging measures associated with general NPSs or the specific subtype after adjustment for age, sex, BMI, and MMSE and CBI scores, along with corrected CBF of the whole brain. The longitudinal associations of baseline nutritional markers with subsequent changes in the severity of NPSs or specific subtypes in patients on the AD continuum were evaluated using generalized linear mixed‐effects models over follow‐up, adjusting for confounding factors. Pairwise comparisons were performed using Bonferroni correction, and multiple comparisons were performed using false discovery rate (FDR) correction. All analyses were performed using R (version 4.2.0; R Foundation for Statistical Computing, Vienna, Austria) and SPSS (version 29.0; SPSS Inc., Chicago, IL, USA). All the graphs were produced using GraphPad Prism 9.5 (www.graphpad‐prism.cn, GraphPad Software Inc., San Diego, CA, USA). A two‐sided *p*‐value < 0.05 was considered statistically significant.

## 3 RESULTS

### 3.1 Patients with NPSs on the AD continuum presented poor nutritional status at baseline

A total of 435 participants, including 61 (14.25%) HCs and 374 (85.75%) patients on the AD continuum (285 with NPSs and 89 without NPSs), were included at baseline. Table 1 presents the baseline clinical, serological, and neuropsychological characteristics of the participants in the three groups.

**TABLE 1:** Baseline clinical and laboratory characteristics of participants among three groups.

Variables	HC (*N* = 61)	AD‐nNPS (*N* = 89)	AD‐NPS (*N* = 285)	*F*/χ2/*K*	*p‐*value^*^	*p‐*value^**^
**Demographics**						
Age, years, M ± SD	61.26 ± 8.04	65.98 ± 7.20	66.72 ± 8.64	10.911	<0.001	1.000
Sex, female, n (%)	14.00 (22.95)	37.00 (41.57)	111.00 (38.95)	6.399	0.041	0.658
BMI, kg/m^2^, M ± SD	23.65 ± 2.44	24.91 ± 3.43	23.60 ± 3.13	6.259	0.002	0.005
WHR, M ± SD	0.86 ± 0.07	0.89 ± 0.08	0.87 ± 0.08	1.587	0.206	0.576
Disease course, ears, median (IQR)	—	1.88 (0.63, 4.00)	2.50 (1.50, 3.54)	−1.712	—	0.087
Education, years, median (IQR)	14.00 (11.50, 16.00)	11.00 (8.00, 12.00)	12.00 (9.00, 13.00)	27.306	<0.001	0.426
Disposable annual income, yuan, median (IQR)	51271 (34893, 80539)	46709 (32903, 79326)	46726 (32903, 81752)	0.615	0.735	0.808
SES, scores, median (IQRs)	11.00 (10.00, 12.00)	10.00 (8.00, 11.00)	10.00 (8.00, 11.00)	16.830	<0.001	0.166
Marital status, married, n (%)	55.00 (90.16)	80.00 (89.89)	247.00 (86.67)	1.023	0.600	0.424
*APOE* ε4 carrier, yes, n (%)	8.00 (13.11)	21.00 (23.60)	94.00 (37.75)	9.269	0.010	0.159
**Medical histories**						
Hypertension, yes, n (%)	14.00 (22.95)	38.00 (43.18)	128.00 (44.91)	10.071	0.007	0.713
Diabetes mellitus, yes, n (%)	7.00 (11.48)	15.00 (17.05)	49.00 (17.19)	1.226	0.542	0.941
Cerebrovascular disease, yes, n (%)	5.00 (8.20)	14.00 (15.91)	53.00 (18.60)	3.989	0.136	0.538
Coronary heart disease, yes, n (%)	8.00 (13.11)	14.00 (15.91)	60.00 (21.05)	2.782	0.249	0.271
Dyslipidemia, yes, n (%)	23.00 (37.70)	38.00 (43.18)	123.00 (43.16)	0.640	0.726	0.997
Current smoking, yes, n (%)	9.00 (14.75)	24.00 (27.27)	57.00 (20.00)	3.529	0.171	0.164
Smoking duration, years, M ± SD	28.67 ± 13.12	29.08 ± 12.94	28.46 ± 11.58	0.022	0.978	0.832
Daily smoking volume, cigarette, M ± SD	11.22 ± 6.83	12.60 ± 8.44	13.88 ± 9.73	0.414	0.662	0.573
Passive smoking history, yes, n (%)	7.00 (11.48)	13.00 (14.61)	56.00 (19.65)	2.965	0.227	0.284
Alcohol consumption, yes, n (%)	19.00 (31.15)	26.00 (9.55)	84.00 (29.47)	0.078	0.962	0.962
**Nutritional biomarkers**						
CONUT, score, median (IQR)	3.00 (3.00, 4.00)	3.00 (3.00, 4.00)	3.00 (3.00, 4.00)	0.412	0.814	0.899
PNI, score, M ± SD	47.48 ± 4.68	48.57 ± 4.23	47.31 ± 3.68	3.443	0.033	0.007
GNRI, score, M ± SD	105.02 ± 7.44	105.02 ± 7.44	102.95 ± 7.48	5.496	0.004	0.023
Vitamin D3 ng/mL, median (IQR)	18.80 (14.25, 24.88)	17.35 (12.75, 22.68)	17.00 (12.40, 22.65)	2.201	0.333	0.513
Vitamin D, ng/mL, median (IQR)	19.45 (15.26, 25.55)	18.28 (13.49, 24.16)	18.35 (13.52, 24.55)	0.985	0.611	0.685
Vitamin B12, pg/mL, median (IQR)	464.00 (360.00, 575.00)	457.00 (363.25, 596.50)	462.50 (321.00, 623.50)	0.350	0.839	0.576
Folic acid, (ng/mL, median (IQR)	8.11 (6.23, 11.85)	6.98 (4.93, 10.53)	6.59 (4.56, 8.74)	6.518	0.038	0.194
Hb, g/L, M ± SD	137.66 ± 16.49	134.43 ± 12.78	130.06 ± 15.48	8.046	<0.001	0.016
Glu, mmol/L, median (IQR)	5.59 (4.77, 6.60)	4.78 (4.43, 5.51)	4.91 (4.47, 5.62)	15.992	<0.001	0.410
Alb, g/L, M ± SD	38.48 ± 3.30	39.66 ± 3.23	39.02 ± 2.99	2.865	0.058	0.084
TC, mmol/L, M ± SD	3.96 ± 0.93	4.44 ± 0.91	4.56 ± 1.04	9.499	<0.001	0.354
TG, mmol/L, median (IQR)	1.33 (1.02, 1.66)	1.09 (0.84, 1.45)	1.04 (0.77, 1.54)	7.219	0.027	0.415
BUN, mmol/L, M ± SD	6.15 ± 1.54	5.66 ± 1.46	5.88 ± 1.56	1.848	0.159	0.236
**Neuropsychological battery**						
MNA, score, median (IQRs)	26.00 (24.25, 27.25)	26.00 (24.00, 27.50)	23.50 (21.00, 25.50)	69.209	<0.001	<0.001
MMSE, score, median (IQR)	28.00 (27.00, 29.00)	26.00 (22.50, 28.00)	23.00 (16.00, 26.00)	98.943	<0.001	<0.001
MoCA, score, median (IQR)	27.00 (26.00, 28.50)	21.00 (18.00, 24.00)	17.00 (10.00, 22.00)	155.245	<0.001	<0.001
NPI, score, median (IQR)	0	0	7.00 (3.00, 16.00)	295.833	<0.001	<0.001
HAMD, score, median (IQR)	4.00 (1.50, 6.50)	3.00 (1.00, 5.00)	7.00 (4.00, 11.00)	71.978	<0.001	<0.001
HAMA, score, median (IQR)	3.00 (1.00, 5.00)	3.00 (1.00, 4.00)	6.00 (3.00, 11.00)	68.673	<0.001	<0.001
DDS, score, median (IQR)	8.00 (7.00, 9.00)	8.00 (7.00, 8.00)	8.00 (7.00, 9.00)	5.804	0.055	0.998
CBI, (score, median (IQRs)	0	0.00 (0.00, 1.00)	9.00 (0.00, 28.00)	127.666	<0.001	<0.001

Abbreviations: AD, Alzheimer's disease; AD‐nNPS, patients without NPSs on the AD continuum; AD‐NPS: patients with NPSs on the AD continuum; Alb, albumin; APOE ε4, apolipoprotein E ε4 allele; BMI, body mass index; CBI, Caregiver Burden Inventory.; CONUT, controlling nutritional status; DDS, dietary diversity score; Glu, glucose; GNRI, geriatric nutritional risk index; HAMA, Hamilton Anxiety Scale; HAMD, Hamilton Depression Scale; Hb, hemoglobin; HC, healthy control; MMSE, Mini‐Mental State Examination; MNA, Mini‐Nutritional Assessment; MoCA, Montreal Cognitive Assessment; NPI, Neuropsychiatric Inventory; NPS, neuropsychiatric symptom; PNI, prognostic nutritional index; SES, socioeconomic status; TC, total cholesterol; TG, triglyceride, BUN, blood urea nitrogen; WHR, waist‐to‐hip ratio.

* R×C diagram χ^2^ test, univariate analysis of variance, or Kruskal–Wallis test was used and *p‐*value < 0.05 set as significant.

** Pairwise comparisons between patients with NPSs and those without NPSs through Bonferroni correction, Mann–Whitney *U* test, or χ^2^ test (corrected *p‐*value < 0.025 was indicated statistical significance).

Patients with NPSs on the AD continuum had lower BMI, PNI, and GNRI scores and lower serum hemoglobin levels than those without NPSs (all *p*’s < 0.05). In addition, patients with NPSs had lower MNA, MMSE, and MoCA scores and higher NPI, HAMD, HAMA, and CBI scores than the other participants (all *p*’s < 0.001).

Compared to the HCs, patients with NPSs on the AD continuum were older (*p* < 0.001), had a lower frequency of female participants (*p* = 0.041), educational levels, and SES scores (both *p*’s < 0.001), a higher frequency of a history of hypertension (*p *= 0.007) and *APOE* ε4 carriers (*p *= 0.010), and lower folic acid levels (*p* = 0.038). However, these factors presented no statistically significant differences between those with or without NPSs on the AD continuum (all *p *> 0.05).

### 3.2 Baseline MNA score was associated with the presence of general NPSs, depression, anxiety, apathy, and appetite/eating disorder subtypes

Low MNA scores at baseline were significantly associated with the presence of general NPSs (odds ratio [OR] = 0.778, 95% confidence interval [CI]: 0.685–0.884, *p*_FDR_ < 0.001), depression (OR = 0.844, 95% CI: 0.815–0.958, *p*_FDR_ = 0.003), anxiety (OR = 0.921, 95% CI: 0.853–0.995, *p*_FDR_ = 0.037), apathy (OR = 0.914, 95% CI: 0.838–0.998, *p*_FDR_ = 0.044), and appetite/eating disorders (OR = 0.806, 95% CI: 0.727–0.894, *p*_FDR_ < 0.001) on the AD continuum after adjusting for age, sex, BMI, MMSE score, and CBI score. Table S2 shows the differences in MNA scores between patients with general NPSs or 12 specific subtypes and those without corresponding subtypes on the AD continuum at baseline.

### 3.3 Increased corrected CBF values of the putamen, VTA, and hypothalamus were associated with baseline and longitudinal changes in four specific NPS subtypes

**FIGURE 2:**
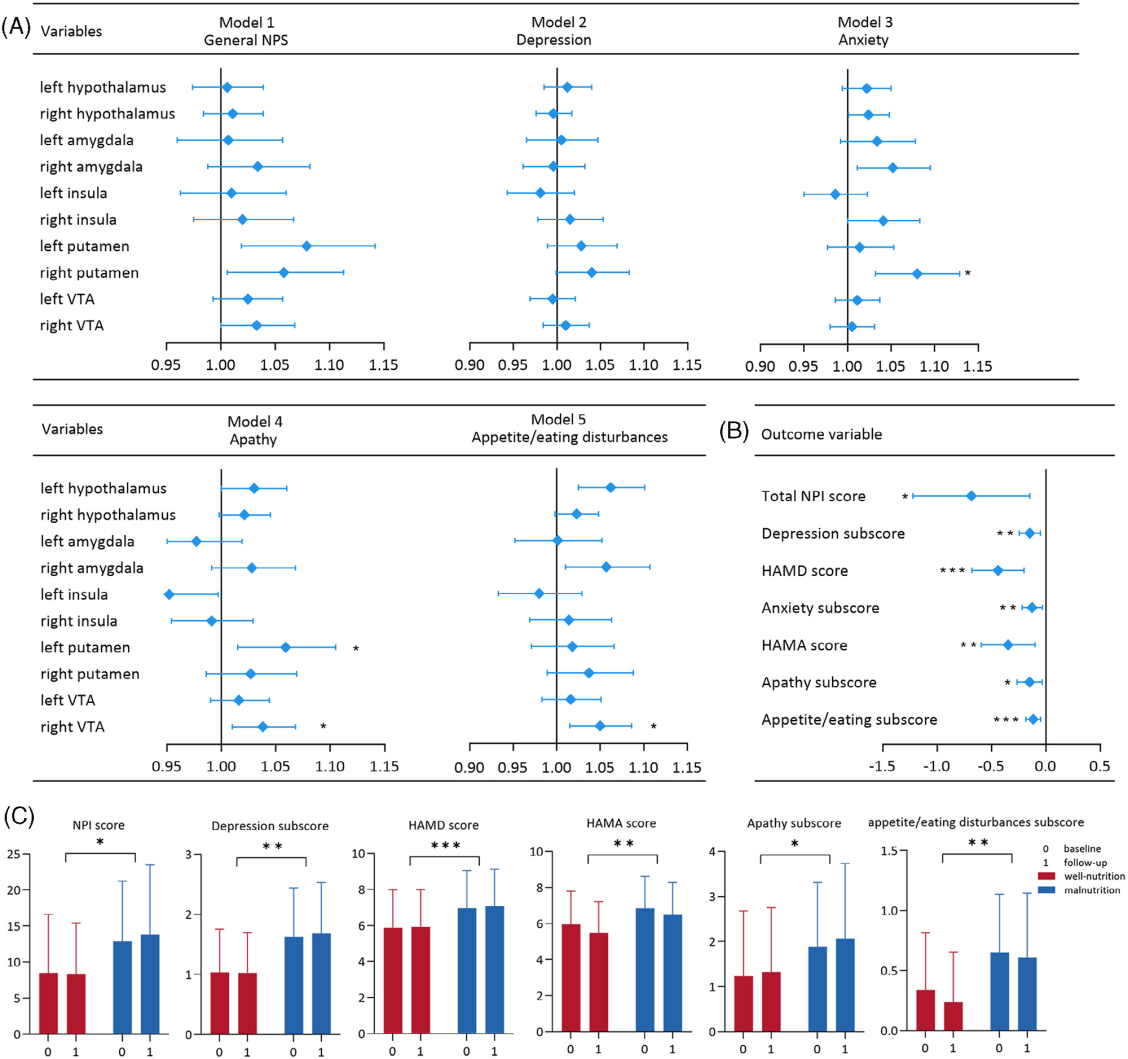
Association of the brain regional corrected CBF or MNA scores with NPSs in patients on the AD continuum. (A) Association between CBF values of the 10 ROIs and NPS subtypes at baseline was assessed by multivariate logistic regression models (*n* = 247). (B) Longitudinal association between MNA score and severity of general NPSs and four subtypes using generalized linear mixed‐effects models (*n* = 136). (C) Association between nutritional status at baseline and longitudinal changes in the severity of general NPSs and four subtypes using generalized linear mixed‐effects models over follow‐up (*n* = 247). *p‐values < 0.05, **p‐values < 0.01, ***p‐values < 0.001 after FDR correction. AD, Alzheimer's disease; CBI, Caregiver Burden Inventory; CBF, cerebral blood flow; FDR, false discovery rate; HAMA, Hamilton Anxiety scale; HAMD, Hamilton Depression Scale; MNA, Mini‐Nutritional Assessment; NPI, Neuropsychiatric Inventory; NPS, neuropsychiatric symptom; ROIs, regions of interest; VTA, ventral tegmental area.

Figure 2A shows the association between the corrected CBF values of the 10 ROIs and NPS subtypes at baseline in multivariate logistic regression analysis after adjusting for age, sex, and CBF values of the whole brain. The increased CBF values in the following regions were associated with the respective conditions: right putamen with anxiety (OR = 1.080, 95% CI: 1.032–1.129, *p*_FDR_ = 0.010); left putamen (OR = 1.059, 95% CI: 1.015–1.105, *p*_FDR_ = 0.040) and right VTA (OR = 1.038, 95% CI: 1.010–18, *p*_FDR_ = 0.040) with apathy; and left hypothalamus (OR = 1.062, 95% CI: 1.025–1.101, *p*_FDR_ = 0.010) and right VTA (OR = 1.050, 95% CI: 1.015–1.086, *p*_FDR_ = 0.020) with appetite/eating disorders.

Among 374 patients on the AD continuum, 136 patients (36.36%) completed follow‐up in an average of 8.79 ± 4.11 months. The clinical profiles of patients lost to follow‐up and those of patients who completed the follow‐up were not significantly different (Table S3). Compared with the patients without NPSs, among patients with NPSs, increasing the baseline corrected CBF values of the left putamen (*β* = 0.135, 95% CI: 0.060–0.210, *p *= 0.001) and right VTA (*β* = 0.069, 95% CI: 0.033–0.105, *p *< 0.001) was associated with subsequent elevation in the specific NPI scores of apathy, whereas those of the left hypothalamus (*β* = 0.062, 95% CI: 0.038–0.086, *p *< 0.001) and right VTA (*β* = 0.028, 95% CI: 0.005–0.051, *p *= 0.016) were associated with subsequent elevation in the specific NPI scores of appetite/eating disturbances during the follow‐up period adjusted for age, sex, whole brain CBF, and follow‐up duration.

### 3.4 Malnutrition was longitudinally associated with the deterioration of general NPSs, depression, anxiety, apathy, and appetite/eating disorder subtypes

During follow‐up, the decrease in MNA score was significantly associated with increased general NPI scores (*β* = −0.685, 95% CI: −1.222 to −0.148, *p *= 0.013); HAMD scores (*β* = −0.440, 95% CI: −0.681 to −0.199, *p *< 0.001); HAMA scores (*β* = −0.347, 95% CI: −0.594 to −0.100, *p *= 0.006); and the NPI sub‐scores of anxiety (*β* = −0.126, 95% CI: −0.220 to −0.032, *p *= 0.009), depression (*β* = −0.147, 95% CI: −0.243 to −0.052, *p *= 0.003), apathy (*β* = −0.149, 95% CI: −0.246 to −0.033, *p *= 0.012), and appetite/eating disturbances (*β* = −0.116, 95% CI: −0.183 to −0.049, *p *= 0.001) adjusted for age, sex, BMI, MMSE scores, CBI scores, and follow‐up duration (Figure 2B).

Patients on the AD continuum with or at risk of malnutrition at baseline were significantly associated with a subsequent increase and accelerated deterioration in the NPI scores of general NPSs (*β* = 3.825, *p *= 0.020; *β* = 0.026, *p* = 0.003); the NPI sub‐scores of depression (*β* = 0.841, *p *= 0.004; *β* = 0.006, *p *< 0.001), apathy (*β* = 0.702, *p *= 0.048; *β* = 0.004, *p *= 0.029), and appetite/eating disturbances (*β* = 0.639, *p *= 0.002; *β* = 0.003, *p *< 0.001); along with HAMD (*β* = 2.781, *p *< 0.001; *β* = 0.019, *p *< 0.001) and HAMA scores (*β* = 2.238, *p *= 0.003; *β* = 0.017, *p *< 0.001) during follow‐up adjusted for age, sex, BMI, MMSE scores, CBI scores, and follow‐up duration (Figure 2C).

### 3.5 Malnourished diet worsened the nutritional status and induced depression‐ and anxiety‐like behaviors in APP/PS1 mice

The nutritional status in nature state of AD mice, ranging from young to old, were established before subjecting them to the malnourished diet intervention (Supplementary Material S11). There was no statistically significant difference in the nutritional characteristics between the 4‐ and 7‐month‐old mice, which were included in our study. In addition, there was no significant difference in the eight serum nutritional biomarkers between the ADSD and ADMD groups (Table S4).

**FIGURE 3:**
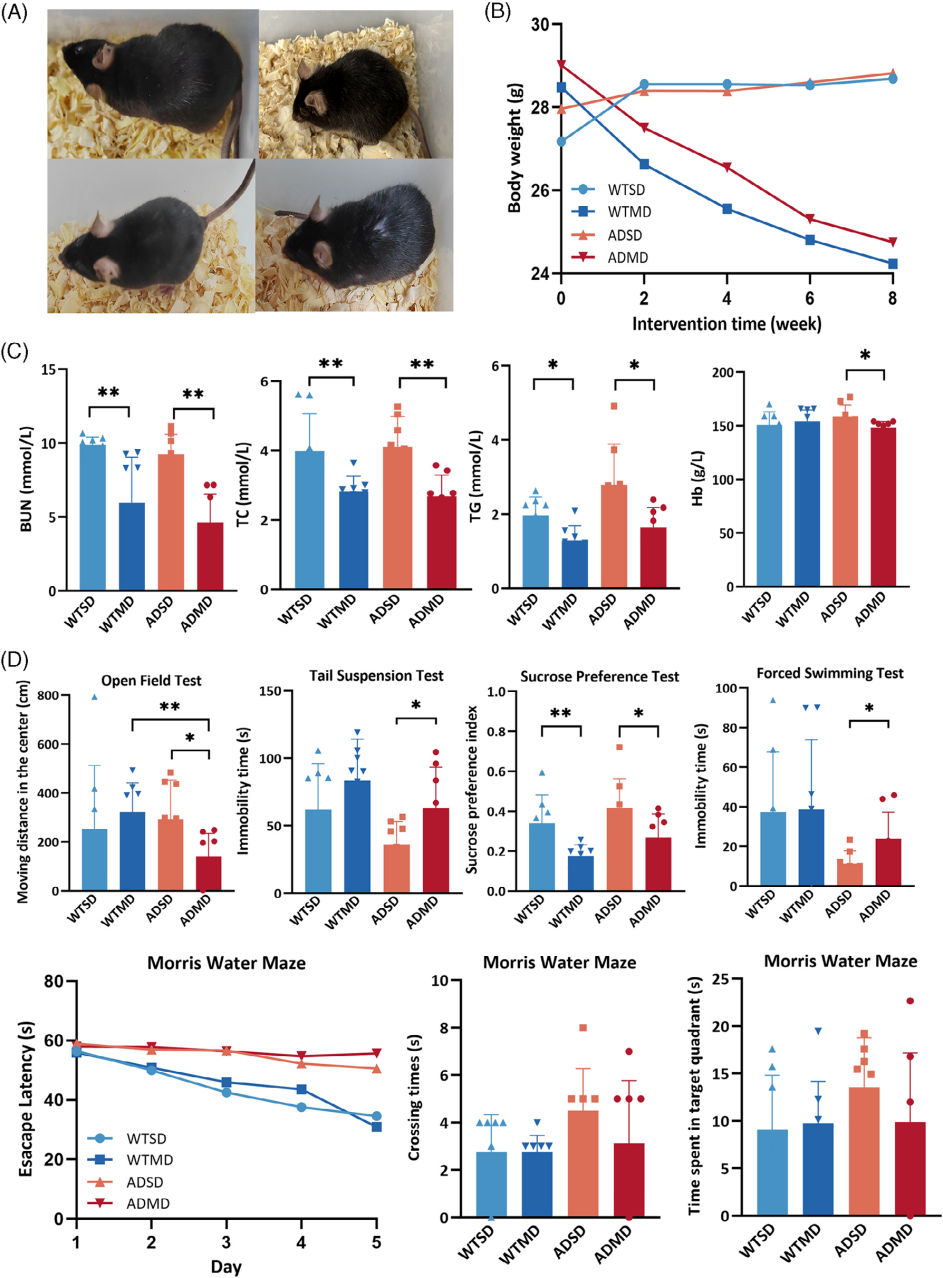
Effects of malnourished diet on nutritional status and NPSs in APP/PS1 mice. (A) Mice fur in the four groups (*n* = 8/group). (B) Changes in the body weight were evaluated through univariate ANOVA or Student's *t*‐test (*n* = 8/group). (C) The serum biomarkers were evaluated using the Kruskal–Wallis or Mann–Whitney *U* tests (*n* = 8/group). (D) The behavioral performances were evaluated using the Kruskal–Wallis or Mann–Whitney *U* tests (*n* = 8/group). *p  < 0.05, **p  < 0.01, ***p  < 0.001. APP/PS1, transgenic APPswe/PSEN1dE9; ADMD, APP/PS1 mice with a malnourished diet; ADSD, APP/PS1 mice with a standard diet; BUN, blood urea nitrogen; FST, forced swim test; Hb, hemoglobin; OFT, open field test; SPT, sucrose‐preference test; TC, total cholesterol; TG, triglyceride; TST, tail suspension test; WTMD, wide‐type mice with a malnourished diet; WTSD, wide‐type mice with a standard diet.

Mice, particularly, APP/PS1 mice, fed a MD exhibited graying and rough fur texture (Figure 3A). The malnourished diet significantly decreased the body weights of both WT (*p* = 0.017) and APP/PS1 mice (*p *= 0.048), compared with those of the mice fed the SD (Figure 3B). Compared with the ADSD group mice, the ADMD group mice had lower levels of serum hemoglobin (*p *= 0.032), BUN (*p *= 0.001), TC (*p *= 0.006), and triglyceride (*p *= 0.016; Figure 3C). The ADMD group mice exhibited a smaller left striatum volume than the ADSD group mice (*t* = 7.277, *p* = 0.018; Figure S1).

Figure 3D shows the behavioral performances of all mice in this study. Compared with mice in the ADSD group, those in the ADMD group showed less moving distance in the central area during the OFT (*p *= 0.004), increased immobility time in the TST (*p *= 0.046), decreased sucrose‐preference values (*p *= 0.043), and decreased time spent floating (*p *= 0.032) in the FST; similar findings were recorded only in the sucrose‐preference test in the WT mice (*p *= 0.007), suggesting malnourished diet‐induced depression‐ and anxiety‐like behaviors in the APP/PS1 mice.

### 3.6 Malnourished diet contributed to the imbalance of dopaminergic‐acetylcholinergic synaptic metabolism in the midbrain and striatum of APP/PS1 mice

**FIGURE 4:**
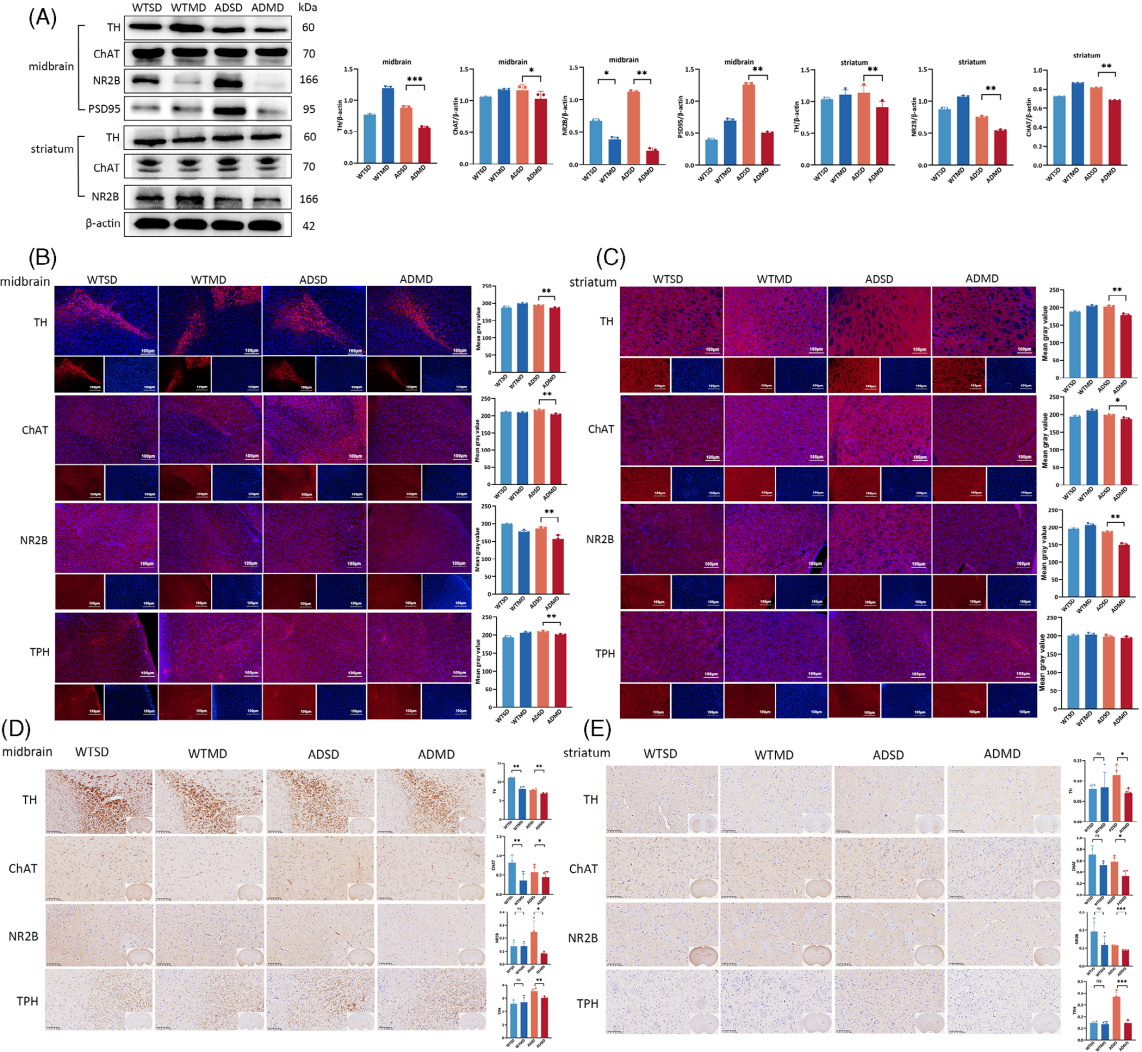
Malnourished diet affects neurotransmitter levels in the midbrain and striatum in APP/PS1 mice. (A) WB and quantification results show a significant decrease in the expression of TH, ChAT, and NR2B in both midbrain and striatum in the ADMD group mice compared to the ADSD group mice (*n* = 3/group). (B) (C) IF staining and quantification show a significant decrease in the expression of TH, ChAT, NR2B in both the midbrain and striatum, and the lower level of TPH in midbrain in ADMD mice relative to ADSD mice (*n* = 3/group); neurotransmitter antibodies (red), DAPI (blue), Scale bar: 100 µm. (D) (E) Immunohistochemical staining of TH, ChAT, NR2B, and TPH in the midbrain and striatum between ADMD and ADSD mice (*n* = 3/group). Scale bar: 100 µm. Data are shown as the mean  ±  SD, and analyzed using Student's *t*‐test, *p  < 0.05, **p  < 0.01, ***p  < 0.001. APP/PS1, transgenic APPswe/PSEN1dE9; ADMD, APP/PS1 mice with a malnourished diet; ADSD, APP/PS1 mice with a standard diet; ChAT, anti‐choline acetyltransferase antibody; IF, immunofluorescence; IHC, immunohistochemistry; NR2B, anti‐*N*‐methyl‐D‐aspartate 2B antibody; PSD95, postsynaptic density protein 95; TH, anti‐tyrosine hydroxylase antibody; TPH, anti‐tryptophan hydroxylase antibody; WB, western blot; WTMD, wide‐type mice with a malnourished diet; WTSD, wide‐type mice with a standard diet.

In three independent experiments, compared with mice in the ADSD group, those in the ADMD group demonstrated reduced levels of bound‐form tyrosine hydroxylase (TH), choline acetyltransterase (ChAT), N‐methyl‐D‐aspartate receptor subunit 2B (NR2B), anti‐tryptophan hydroxylase antibody (TPH), and postsynaptic density 95 (PSD95) expression in the midbrain, and reduced levels of TH, ChAT, NR2B, and TPH expression in the striatum (all *p *< 0.05; Figure 4A–E). However, only NR2B expression in the midbrain using WB, and the TH and ChAT levels in the midbrain using IHC were lower in the WTMD group mice than in the WTSD group (all *p*’s < 0.05; Figure 4A–E).

**FIGURE 5:**
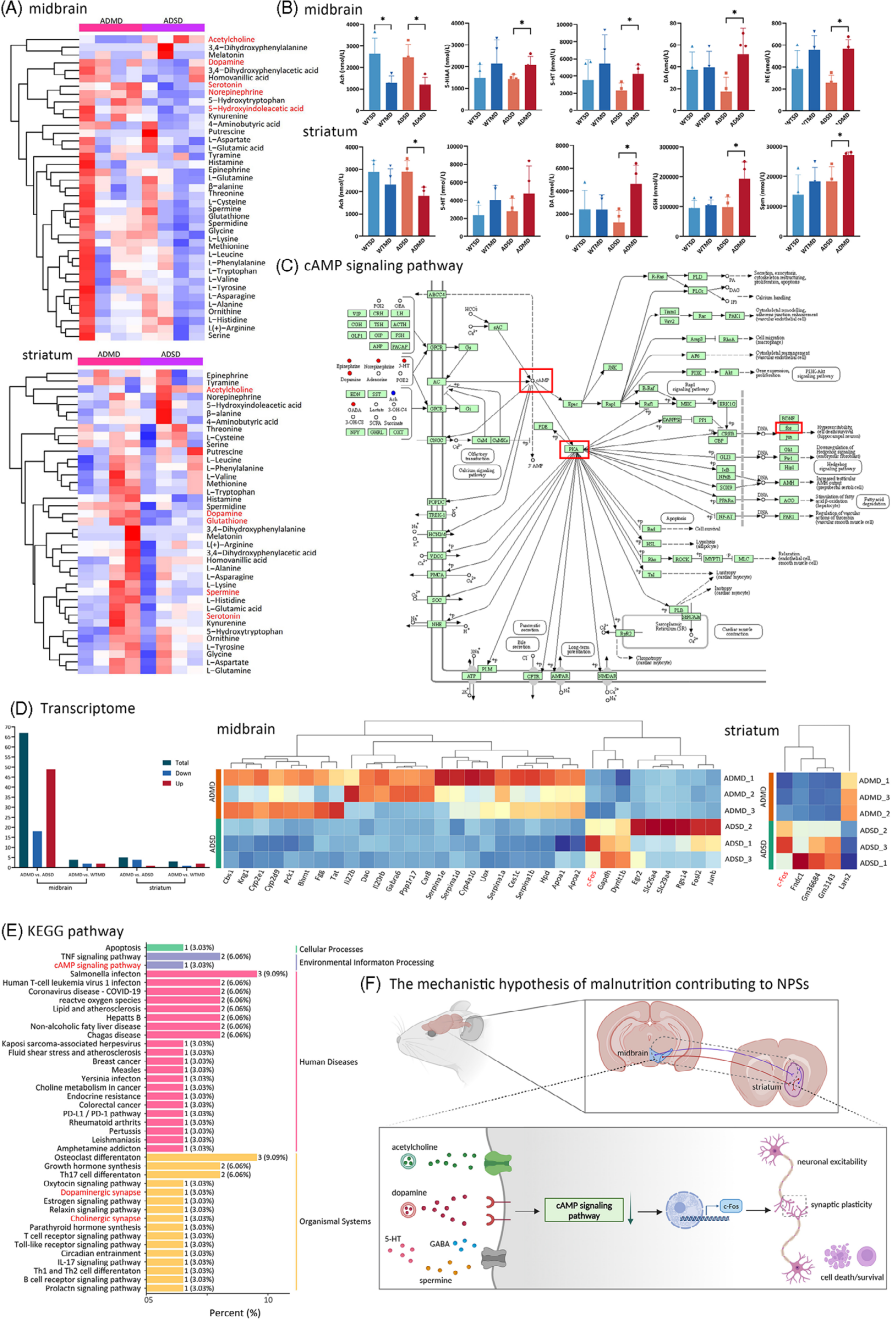
Integrated metabolomic and transcriptomic analyses reveal shared cAMP enrichment pathway. (A), (B) Heatmap and quantification results show the effect of a malnourished diet on the levels of neurotransmitters in both the midbrain and striatum of APP/PS1 mice using Student's *t*‐test (*n* = 4/group). (C) The main differential neurotransmitters were enriched in the cAMP signaling pathway based on KEGG database analysis. (D) Heatmap showing the expression levels of DEGs in the midbrain and striatum transcriptomes between ADSD and ADMD mice (*n* = 4/group). (E) KEGG pathway of *c‐Fos* gene shows significantly enriched *c‐Fos* expression in the cAMP signaling pathway. (F) The mechanistic hypothesis of how malnutrition contributes to the occurrence of NPSs. APP/PS1, transgenic APPswe/PSEN1dE9; ADMD, APP/PS1 mice with a malnourished diet; ADSD, APP/PS1 mice with a standard diet; cAMP, cyclic adenosine monophosphate; DEGs, differentially expressed genes; KEGG, Kyoto Encyclopedia of Genes and Genomes; NPSs, neuropsychiatric symptoms; WTMD, wide‐type mice with a malnourished diet; WTSD, wide‐type mice with a standard diet.

A total of 39 neurotransmitter compounds have been identified in the midbrain and striatum. Among them, we recorded significantly decreased levels of acetylcholine in both the midbrain (*p *= 0.010) and striatum (*p *= 0.014), increased levels of 5‐hydroxyindoleacetic acid (*p *= 0.028), 5‐HT (*p *= 0.033), dopamine (*p *= 0.045), and norepinephrine (*p *= 0.028) in the midbrain, and increased levels of dopamine (*p *= 0.013), glutathione (*p *= 0.027), and spermine (*p *= 0.013) in the striatum (Figure 5A,B) were in the ADMD group mice relative to the ADSD group. Thus, a malnourished diet induces an imbalance in dopaminergic‐acetylcholinergic synaptic metabolism and neurotransmitter activity in the midbrain and striatum of APP/PS1 mice.[Fig alz14506-fig-0006]

KEGG analyses yielded 60 signaling pathways in both striatum and midbrain (*Q*‐value < 0.05), mainly including synaptic vesicle cycle, mineral absorption, glutathione metabolism, and cAMP signaling pathway, as well as alanine, aspartate, and glutamate metabolism. Table S5 presents the *Q*‐values of all enriched metabolic pathways of differential neurotransmitters in both striatum and midbrain based on the KEGG database. Five main differential neurotransmitters in both the midbrain and striatum, including dopamine, acetylcholine, norepinephrine, GABA, and 5‐HT, were enriched in the cAMP signaling pathway, which was related to synaptic plasticity and neuronal excitability (Figure 5C).

### 3.7 RNA sequencing revealed the significantly downregulation of *c‐Fos* gene expression in the midbrain and striatum of APP/PS1 mice

Table S6 presents all the DEGs in the midbrain and striatum of mice between the ADMD group and ADSD group. A total of 67 significant DEGs were identified in the midbrain of mice from the ADMD group compared with those in mice from the ADSD group (*p_FDR_*  < 0.05), of which 49 genes were upregulated and 18 genes were downregulated. Five genes were significantly differentially expressed in the striatum of mice from the ADMD group (*p_FDR_*  < 0.05), of which four genes were downregulated, and only one gene was upregulated (Figure 5D,E). Of the DEGs, *c‐Fos* was the only shared downregulated gene in both the midbrain and striatum in APP/PS1 mice fed a malnourished diet (Figure 5F). Table S7 presents the *Q*‐values of all enriched metabolic pathways associated with *c‐Fos* in both the striatum and midbrain based on the KEGG database. The *c‐Fos* gene was categorized in the 44 main pathways by the KEGG database, including the cAMP signaling pathway and dopaminergic and cholinergic synapses. Table S8 presents the *Q*‐values of 58 enriched pathways associated with *c‐Fos* in both the striatum and midbrain based on the GO database. Notably, the *c‐Fos* and aforementioned differential neurotransmitters were enriched in the cAMP signaling pathway, suggesting that this pathway could be a potential signaling pathway associated with both malnutrition and NPS‐like behaviors (Figure 5F).

### 3.8 Downregulation of the cAMP signaling pathway in the midbrain and striatum was influenced by malnutrition and associated with the worsened NPSs in APP/PS1 mice

**FIGURE 6 alz14506-fig-0006:**
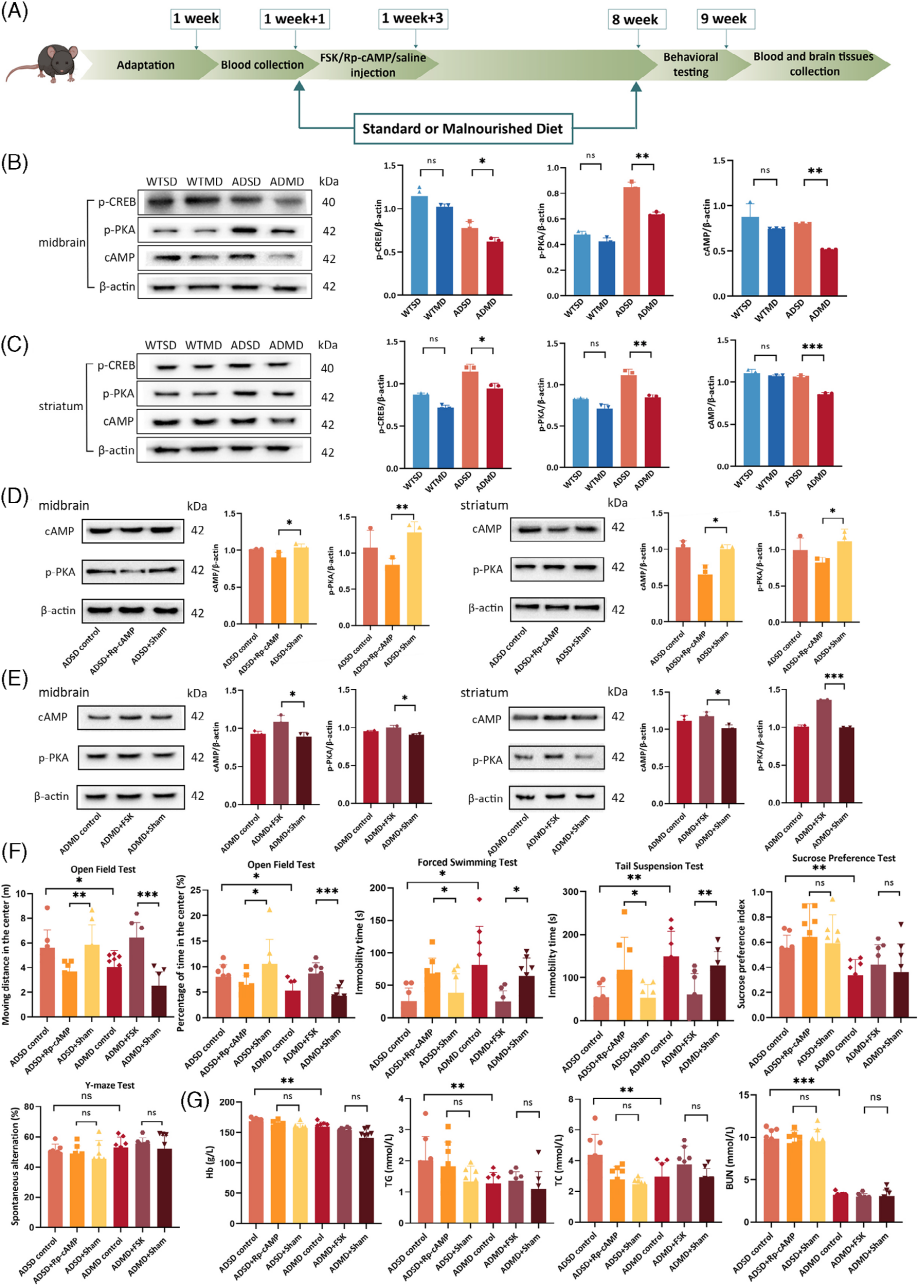
Downregulation of the cAMP signaling pathway–related components in the midbrain and striatum influenced by malnutrition and associated with worsening of NPSs in APP/PS1 mice. (A) Schematic diagram of independent pharmaceutical in vivo validation experiments. (B), (C) WB presents the cAMP, p‐PKA, p‐CREB expression levels in the midbrain and striatum among four groups after 2‐month diet intervention (*n* = 3/group). (D) WB presents the cAMP and p‐PKA expression levels in the midbrain and striatum among three ADSD groups (*n* = 3/group). (E) WB presents the cAMP and p‐PKA expression levels in the midbrain and striatum among three ADMD groups (*n* = 3/group). (F) Behavioral testing among six groups after a 2‐month diet intervention with independent pharmaceutical experiments (*n* = 8/group). (G) Four serum nutritional biomarker levels among six groups after 2‐month diet intervention with independent pharmaceutical experiments (*n* = 8/group). *p  < 0.05, **p  < 0.01, ***p  < 0.001. APP/PS1, transgenic APPswe/PSEN1dE9; ADMD, APP/PS1 mice with a malnourished diet; ADSD, APP/PS1 mice with a standard diet; BUN, blood urea nitrogen; cAMP, cyclic adenosine monophosphate; FSK, forskolin; Hb, hemoglobin; NPSs, neuropsychiatric symptoms; p‐CREB, phosphorylated cAMP‐responsive element binding protein; p‐PKA, phosphorylated protein kinase A; Rp‐cAMP, a specific inhibitor of cyclic adenosine monophosphate signaling pathway; TC, total cholesterol; TG, triglyceride; WB, western blot; WTMD, wide‐type mice with a malnourished diet; WTSD, wide‐type mice with a standard diet.

The schematic diagram of independent pharmaceutical in vivo validation experiments is shown in Figure 6A. After an 8‐week dietary intervention, the cAMP, p‐PKA, and p‐CREB expression levels decreased significantly in both the midbrain and striatum of ADMD group mice compared with those in the midbrain and striatum of the ADSD group mice (all *p_FDR_* < 0.05; Figure 6B,C).

Notably, compared with ADSD+Sham group mice, ADSD+Rp‐cAMP group mice showed significantly decreased moving distance and percentage of time in the central area during OFT (*p *= 0.004; *p *= 0.037), and increased immobility time in the TST and time spent floating in the FST (*p *= 0.041; *p *= 0.042). Accordingly, compared with ADMD+Sham group mice, those in the ADMD+FSK group showed significantly increased moving distance and percentage of time in the central area during OFT (both *p *< 0.001), and decreased immobility time in the TST and time spent floating in the FST (*p *= 0.005; *p *= 0.004; Figure 6D).

Regarding serum nutritional biomarkers, although the serum levels of hemoglobin, TC, triglyceride, and BUN were significantly decreased in ADMD group mice relative to ADSD group mice (all *p *< 0.05), there was no statistically significant difference in these serum nutritional biomarkers of mice between the ADSD+Sham and ADSD+Rp‐cAMP groups, as well as between ADMD+Sham and ADMD+FSK groups (all *p*’s > 0.05; Figure 6E).

In addition, compared with ADSD+Sham group mice, those in the ADSD+Rp‐cAMP group presented significantly reduced cAMP and p‐PKA expression in both the midbrain and striatum (all *p*’s < 0.05; Figure 6F); compared with the ADMD+Sham group mice, those in the ADMD+FSK group demonstrated increased expression levels of cAMP and p‐PKA in both the midbrain and striatum (all *p*’s < 0.05; Figure 6G).

## 4 DISCUSSION

This prospective cohort study and animal experiments found that poor nutritional status was longitudinally associated with deterioration in general NPSs and four specific NPS domains, including depression, anxiety, apathy, and appetite/eating disturbances in patients on the AD continuum and APP/PS1 mice. Moreover, baseline hyperperfusions in the putamen and VTA were associated with the exacerbation of anxiety, depression, and apathy at both baseline and follow‐up. Furthermore, a malnourished diet contributed to malnutrition and depression‐ and anxiety‐like behaviors in APP/PS1 mice, which was associated with imbalances in the dopamine and acetylcholine levels and downregulation of the cAMP/c‐Fos signaling pathway in both the midbrain and striatum. These findings elucidated the association between malnutrition and NPSs in the AD continuum and provided preliminary evidence revealing the relevance of the cAMP signaling pathway in this association; thus, we underscore the potential nutritional intervention‐related targets that can be used for the formulation of NPS management strategies.

Current data are insufficient to establish a clear link between nutrition and NPSs in patients on the AD continuum.^32^, ^33^ Our study identified baseline and long‐term poor nutritional status as risk predictors for subsequent deterioration in general NPSs, depression, apathy, anxiety, and appetite/eating disturbances during follow‐up. Similarly, a longitudinal study revealed that women with malnutrtion or at risk of malnutrition were significantly associated with increased NPS severity in MCI and early‐stage AD during a 2.5‐year follow‐up.^14^ A retrospective study including 3299 older patients found that the highest prevalence of (risk of) malnutrition was observed in patients with dementia or depression.^34^ Another retrospective study found higher malnutrition risk was significantly associated with anxiety symptoms in patients with cancer.^35^ Moreover, a cross‐sectional study including 271 people aged ≥60 years found that malnutrition was associated with depression.^36^ Poor nutritional status is related to an unhealthy dietary pattern and AD disease progression^1^, ^37^; however, similar to recent findings, dietary patterns are not related to malnutrition in different stages of dementia and normal cognition.^38^ Furthermore, after fully considering confounding factors, including cognitive decline and disease progression, we still found that malnutrition was associated with NPSs in patients on the AD continuum. It highlights the potential longitudinal impact of clinical nutritional status on altered NPSs, suggesting that clinicians should consider and manage malnutrition in the early stages of AD to prevent the onset and development of NPSs.

Our findings demonstrate that poor nutritional status affects the onset and development of NPSs, focusing on affective symptoms (depression, apathy, and anxiety). A potential reason for this is that affective symptoms and dietary nutrition may share common regulatory brain regions, and the impairment of these regions could affect neurotransmitter release along with nutrient intake and absorption.^17^, ^39^ The hypothalamus is the “master orchestrator” for the central regulation of energy balance, dieting, and metabolism. For instance, the lateral hypothalamus, from where anxiogenic brain signals originate, is sensitive to severe food deprivation and nutritional status and is critical for generating context‐appropriate actions.^40^ Our study found that increased CBF values in the left hypothalamus led to the onset and development of appetite/eating disturbances in patients on the AD continuum. However, the relationship between the hypothalamus and affective symptoms remains unclear, suggesting that the hypothalamus, as a key integrating center involved in regulating nutrient homeostasis, is more likely to be associated with appetite/eating disturbances than with affective symptoms.^41^ Apart from the hypothalamus, diverse cortico‐limbic structures, which are substrates for adaptive behavioral and emotional responses, are also involved in homeostatic regulation and “hedonic” control of food intake.^24^, ^42^ In the present study, we found that increasing baseline hyperperfusion of the right putamen was associated with anxiety, whereas baseline hyperperfusion of the left putamen and right VTA was associated with subsequent deterioration of apathy. Similarly, a meta‐analysis of neuroimaging studies found that gray‐matter atrophy and dysfunctional connectivity in the putamen are associated with apathy.^43^ Another meta‐analysis showed that putamen atrophy in AD was associated with apathy, potentially independent of cognitive impairment and depression.^44^ A single‐photon emission computed tomography (SPECT)–based study also found dopaminergic neuronal loss in the bilateral putamen in patients with apathy, including those with AD and dementia with Lewy bodies.^45^ Moreover, hyperperfusion of the right VTA was associated with subsequent deterioration of apathy and appetite/eating disturbances. A SPECT‐based study on 59 patients with early AD found that the severity of apathy was inversely correlated with regional CBF in the midbrain.^46^ In the MCI stage, several functional, structural, and metabolic alterations affecting VTA dopaminergic neurons correlate with NPS appearance.^47^ A cross‐sectional study revealed that the degree of atrophy in the mesocorticolimbic dopamine‐secreting regions of the brain positively correlates with the severity of depression, anxiety, and apathy in MCI to AD and AD dementia subgroups.^48^ Together, these findings partially support our results and provide novel insights into the dopaminergic pathway, which involves the putamen and VTA; this may represent a potential mechanism underlying the onset and development of NPSs, particularly, affective symptoms.

Our findings from animal models further demonstrate that a malnourished diet contributed to the anxiety‐ and depression‐like behaviors in APP/PS1 mice and was associated with neurotransmitter imbalance and the downregulation of *c‐Fos* gene expression in the midbrain (VTA) and striatum (putamen). Consistently, an imbalance in multiple neurotransmitter pathways causes depression‐like behavior in APP/PS1/tau triple transgenic mice.^49^ A trigeminal neuralgia mouse study found that the overactivity of dopaminergic neurons in the VTA underscored the depressive behavior that developed under a state of chronic neuropathic pain.^50^ Another animal study showed that exercise improved neuropsychiatric disorders, including depression and anxiety, by enhancing striatal dopamine release.^51^ Furthermore, c‐Fos, an immediate early gene product, is transiently expressed following neural activation.^52^ This aligns with a recent rat study demonstrating that morphine (1.25/1.5 µg) injections into the pedunculopontine tegmental nucleus significantly reduce feeding behavior induced by electrical VTA stimulation, which corresponds with altered neuronal activity (c‐Fos protein) in most brain structures.^53^ Alcohol withdrawal drives depressive behaviors and is accompanied by significantly upregulated *c‐Fos* gene expression in VTA dopaminergic neuron–projecting rostromedial tegmental nucleus GABA neurons.^54^ A very recent study also found that hedonic feeding in the absence of hunger elevated c‐Fos expression in a subset of DBBPenk neurons of male mice.^55^ It suggests that *c‐Fos* expression is related to dietary nutrition and synaptic plasticity and neurotransmitter release, especially in the midbrain.

Notably, KEGG pathway analysis based on our transcriptome and metabolome findings and independent pharmaceutical experiments demonstrated that the downregulation of the components of the cAMP signaling pathway in the midbrain and striatum was influenced by malnutrition and associated with the worsening of NPSs. The cAMP signaling pathway is an important regulator that mediates intracellular responses to multiple hormones and neurotransmitters.^56^ Dysregulation of the Ca^2+^/cAMP signaling pathway may be an upstream issue that links to obesity and depression by controlling the release of both neurotransmitters and hormones,^57^ suggesting a connection between nutritional status and NPSs. Reduction in the cAMP/PKA cascade potentially affects neuronal excitation and synaptic plasticity, ultimately leading to depression, whereas activation of the cAMP/PKA cascade would provide a rapid antidepressant effect.^58^ Accordingly, a recent review also emphasized the central roles of cAMP/PKA signaling in anxiety.^59^ The APP/PS1/tau mice treated with rolipram for 10 days show a significant attenuation of anxiety‐ and depression‐like behaviors by increasing cAMP/PKA signaling and decreasing neuroinflammation.^60^ In our study, cAMP signaling pathway inhibition or activation did not change the serum nutritional biomarkers levels but significantly changed the NPS‐like behavior, suggesting cAMP signaling pathway mediation in the association between malnutrition and NPSs.

This study has a few limitations. First, some patients were lost to follow‐up due to coronavirus disease, which resulted in a relatively small follow‐up sample size; however, no significant difference was observed between the baseline clinical characteristics of those lost to follow‐up and those who completed follow‐up. Second, considering the complexity, high heterogeneity, and dynamic changes of NPSs, it is difficult to include all the brain regions or single out which brain regions are the main source responsible for the nutritional status‐associated NPS. Thus, our study explores only the potential role of the midbrain and striatum on the association between malnutrition and NPSs based on our previous evidence. Finally, this study preliminarily suggests that the downregulation of cAMP signaling pathway in the midbrain and striatum was influenced by malnutrition and associated with the worsening of NPSs; however, the exact mechanisms and causal role of the cAMP signaling pathway in malnutrition‐induced exacerbation of NPSs require further validation.

In conclusion, our study showed that in both patients with AD and APP/PS1 mice, poor baseline nutritional status was associated with subsequent deterioration of NPSs, particularly, depression, apathy, and anxiety, over a follow‐up period. Moreover, baseline hyperperfusion of the midbrain and striatum was associated with subsequent deterioration of general NPSs and apathy in patients on the AD continuum. Thus, clinicians should consider malnutrition on admission and strive to prevent and manage NPSs in patients with AD. Furthermore, we found that malnutrition‐worsened affective symptoms in APP/PS1 mice was associated with the downregulation of cAMP signaling pathway in both the midbrain and striatum. These findings provide novel insights into nutritional intervention targets for NPSs at an early stage. Future research, including translational studies, is needed to elucidate the exact mechanism and causal role of cAMP signaling pathway in inducing the development of NPSs in different AD animal models, and prospective studies and clinical trials to identify the best strategies to prevent and alleviate NPSs via targeting the cAMP signaling pathway in patients on the AD continuum.

## AUTHOR CONTRIBUTIONS

Jiwei Jiang designed the study, performed the cohort study, and wrote and revised the manuscript. Tianlin Jiang, Min Zhao, Xiaohong Wang, Huiying Zhang, and Jiawei Zhou performed animal experiments. Hanping Shi revised the manuscript. Jiwei Jiang, Tianlin Jiang, Wenyi Li, and Xiaoli Zhang analyzed the data. Tianlin Jiang and Shirui Jiang drew the figures. Qiwei Ren, Linlin Wang, and Shiyi Yang collected the clinical data. Zeshan Yao and Yaou Liu performed imaging acquisition and segmentation. Jun Xu designed the study and revised the manuscript, and also aided in study screening procedures and funding. All authors critically reviewed the manuscript, agreed to be fully accountable for ensuring the integrity and accuracy of the work, and read and approved the final manuscript before submission.

## CONFLICT OF INTEREST STATEMENT

All authors declare no conflicts of interest. Author disclosures are available in the supporting information.

## ETHICS STATEMENT

The human study included participants from the Chinese Imaging, Biomarkers, and Lifestyle (CIBL) study, which was approved by the ethics committee of Capital Medical University, Beijing Tiantan Hospital (approval number: KY‐2021‐028‐01) and registered at chictr.org.cn (ChiCTR2100049131). All participants or their legally authorized caregivers (if necessary) provided written informed consent for participation and publication. The animal study was performed in accordance with the regulations of the Animal Experiment Ethics Committee of Yangzhou University (YXYLL‐2022‐71).

## Supporting information



Supporting Information

Supporting Information

## Data Availability

The data supporting the conclusions of this article will be made available upon a request sent by email to the corresponding author.
