# Supplementary material for: Malnutrition exacerbating neuropsychiatric symptoms on the Alzheimer's continuum is relevant to the cAMP signaling pathway: Human and mouse studies
Source: Alzheimers Dement. 2025 Jan 27;21(2):e14506. doi: 10.1002/alz.14506 (PMC11848410; doi:10.1002/alz.14506)
Supplement: Supplementary file 1 — Supporting Information [file ALZ-21-e14506-s002.docx]

**Malnutrition exacerbates neuropsychiatric symptoms in Alzheimer's continuum by suppressing cAMP signaling pathway: human and mouse studies**

**Contents**

S01. The detailed inclusion and exclusion criteria in the human cohort study……………………………….2

S02. The assessment of socioeconomic status…………………………………….…………………………………….….3

S03. The detailed calculation formulas for three objective nutritional tools….………………………………4

S04. The detailed comprehensive neuropsychological and nutritional assessments……………..………5

S05. The detailed parameters and processing of multimodal neuroimaging…………………………......…6

Structural 3D-T1 weighted imaging…………………………………………………………………………….......…6

7-delay pCASL…………………………………………………………………………………………………………………....6

S06. The composition of malnourished and standard diet…………………………………………………..……....7

S07. The detailed process of behavioral testing in the mice study..…………………………………..………….8

S08. The detailed protocols of western blot, immunofluorescence, and immunohistochemistry....9

Table S1. Details of primary antibodies used in western blot, immunofluorescence, and immunohistochemistry…………………………………………………..………………………………………………………...…..11

S09. Quasi-targeted metabolomics analysis…………………………………………………..…………………..……...12

S10. Transcriptomic analysis of brain tissues…………………………………………………..…………………..………13

Table S2. The baseline differences of MNA score between patients with NPS or its specific sub-type and those without………………………………………………………………………………………………………………………….15

Table S3. The baseline clinical profiles of patients on the AD continuum with follow-up and those lost to follow-up…………………………………………………………………………………………………………………………….16

S11. The nutritional status of AD mice, ranging from young to old, before malnutrition treatment……………………….………………………………………………………………………………………………………………18

Table S4. The nutritional biomarkers among the four groups at baseline……………………………………..19

Figure S1. Structural T2-weighted imaging of the four groups after two-month dietary intervention……………………………………………………………………………………………………………………………………20

Table S5. The Q values of all enriched metabolic pathways of differential neurotransmitters in both midbrain and striatum based on the KEGG database………………………………………………………..…………...21

Table S6. All the differentially expressed genes in midbrain and striatum of mice between ADMD group and ADSD group…………………………………………………………………………………………………………………..24

Table S7. The Q values of all enriched metabolic pathways of *c-Fos* in both midbrain and striatum based on the KEGG database………………………………………………………………………………………………………...26

Table S8. The Q values of all enriched pathways of *c-Fos* in both midbrain and striatum based on the GO database…………………………………………………………………………………………………………………………….28

References………………………………………………………………………………………………………………………………….30

# S01. The detailed inclusion and exclusion criteria in the human cohort study

The inclusion criteria were as follows: participants (1) aged 50 to 90 years; (2) were right-handed; (3) patients with mild cognitive impairment (MCI) were diagnosed based on 2018 National Institute on Aging Alzheimer’s Association (NIA-AA) workgroup diagnostic criteria [[1](#_ENREF_1)]; (4) patients with AD dementia (age of onset ≥ 60 years) met the 2018 NIA-AA workgroup or 2024 AA workgroup diagnostic criteria for confirmed AD dementia [[1](#_ENREF_1), [2](#_ENREF_2)]; (5) could cooperate to complete comprehensive neuropsychological assessments.

The exclusion criteria were as follows: patients with (1) cognitive impairment due to other etiology, including other central neurodegenerative diseases (including dementia with Lewy bodies, frontotemporal dementia, or Parkinson's disease), concomitant central nervous system diseases (such as cerebrovascular disease, tumor, encephalitis, and epilepsy), and traumatic brain injury; (2) a history of mental disorders, including bipolar disorder and schizophrenia, in accordance with the Diagnostic and Statistical Manual of Mental Disorders-5 [[3](#_ENREF_3)]; (3) a history of excessive drinking, drug abuse, or exposure to toxic materials within the past 2 years; or (4) had systemic diseases, such as syphilis or human immunodeficiency virus infection.

All healthy controls were ≥ 50 years of age, cognitively normal {without subjective or objective memory complaints, Mini-Mental State Examination (MMSE) score of 26–30, and Montreal Cognitive Assessment (MoCA) score of 24-30}, and free of any general or laboratory evidence of other diseases that could impact cognition and neuropsychiatric symptoms.

**S02. The assessment of socioeconomic status [**[**4**](#_ENREF_4)**]**

| Variable | Definition and assignment |
| --- | --- |
| Education level | 1 = Illiterate/Primary school, 2 = Junior high school, 3 = Senior high school/Vocational high school/Technical secondary school, 4 = Junior college/University, 5 = Postgraduate and higher |
| Income level | Annual per capita household income = Total annual household income/Household size. 1 = Less than 10,000 yuan; 2 = 10,000– 29,999 yuan; 3 = 30,000–49,999 yuan; 4 = 50,000–69,999 yuan; 5 = 70,000 yuan and higher |
| Professional status | 1 = The unemployed/Retiree; 2 = Farmer/Worker; 3 = Enterprise employee/Personnel of other public institutions/Businessman/ College student; 4 = Teacher/Medical staff; 5 = Civil servant |
| Socioeconomic status | The individual’s comprehensive socioeconomic status is measured by adding the scores of education level, income level and professional status. The higher the score is, the higher the status is. |

**S03. The detailed calculation formulas for three objective nutritional tools**

The CONUT score included 3 variables (serum albumin, total cholesterol, and total lymphocyte count) to evaluate nutritional risk in hospitalized patients [[5](#_ENREF_5)]. The higher the score, the worse the nutritional status. Scores of 0 to 1, 2 to 4, 5 to 8, 9 to 12 were considered normal, mild, moderate, and severe malnutrition risk, respectively.

The PNI was calculated using the formula: 10×serum albumin (g/dl) +0.005×total lymphocyte count (mm^3^) [[6](#_ENREF_6)]. The lower the score, the worse the nutritional status. PNI scores >38, 35 to 38, <35 were considered as normal, moderate, and severe malnutrition risk, respectively.

The GNRI was calculated as (1.519×serum albumin [g/L] +41.7×present weight [kg]/ideal body weight [kg]) [[7](#_ENREF_7)]. The ideal body weight was calculated according to the Lorentz equations as follows: for men: ideal body weight=height in cm−100- ([(height in cm−150]/4); for women: ideal body weight=height in cm−100−([height in cm−150]/2). The lower the score, the worse the nutritional status. GNRI scores >98, 92 to 98, 82 to 91, <82 were considered normal, mild, moderate, and severe malnutrition risk, respectively.

# S04. Detailed comprehensive neuropsychological and nutritional assessments

NPSs and relevant sub-symptoms were evaluated using the Neuropsychiatric Inventory (NPI), a fully structured interview for investigating 12 different behavioral and neuropsychiatric domains, including delusion, hallucination, agitation, depression, anxiety, euphoria, apathy, disinhibition, irritability, aberrant motor activity, sleep and nighttime behavior, and appetite or eating disturbances [[8](#_ENREF_8)]. The NPI provides a severity score on a 3–point scale and a frequency score on a 4–point scale for each specific subtype. The total NPI severity score is calculated by summing the 12 severity sub–scores (severity score × frequency score). The higher the general or specific domain score of NPI, the worse the general or specific NPS.

Considering the depression and anxiety are the most common sub-symptoms of NPS on the AD continuum, the severity of depression and anxiety was also assessed by the 17-item Hamilton Depression Scale (HAMD-17) [[9](#_ENREF_9)] and 14-item Hamilton Anxiety Scale (HAMA-14), respectively [[10](#_ENREF_10)]. The higher the HAMD-17 and HAMA-14 scores, the worse the depression and anxiety.

Global cognition was evaluated using the Chinese version of the Mini-Mental State Examination (MMSE) [[11](#_ENREF_11)] and Beijing version of the Montreal Cognitive Assessment (MoCA) [[12](#_ENREF_12)]. They usually include seven cognitive domains, including visuospatial and executive function, naming, attention, abstraction, language, delayed memory, and orientation. Patients with a total MMSE score of ≤24, ≤20, and ≤17 for >6, 1–6, and 0 years of education, respectively, or a total MoCA score of ≤24, ≤20, and ≤14 for >6, 1–6, and 0 years of education, respectively, were considered to have cognitive impairment.

The caregiver burden was assessed by the Caregiver Burden Inventory (CBI), which is a 24-item multidimensional questionnaire that quantifies the impact of caregiver burden using five domains: time dependence and developmental, physical, social, and emotional burden [[13](#_ENREF_13)]. The total CBI score ranges from 0 to 96, with higher scores indicating greater levels of perceived burden.

The Mini-Nutritional Assessment (MNA), which comprises anthropometric measurements, a global assessment, dietary questionnaire, and subjective assessment, were used to determine the presence and degree of malnutrition [[14](#_ENREF_14)]. The total MNA score can be used to distinguish between well-nourished patients (≥24), patients at risk of malnutrition (17–23.5), and malnourished patients (<17). The higher the MNA score, the better the nutritional status.

The dietary diversity score (DDS) was measured at baseline based on a well validated [food frequency questionnaire](https://www.sciencedirect.com/topics/medicine-and-dentistry/food-frequency-questionnaire) [[15](#_ENREF_15)]. Participants were asked how often they consumed various food items, such as [grain](https://www.sciencedirect.com/topics/food-science/cereal), oil, meat, fish, eggs, beans, salted vegetables, garlic, tea, fresh vegetables, and fruits, all of which are traditionally included in the Chinese diet. We did not include grain or oil in the construction of the DDS because almost all Chinese individuals consumed these 2 foods every day, similar to previous studies [[16](#_ENREF_16)]. Response options on these food item questions included “almost every day,” “occasionally,” and “rarely or never.” If the response for 1 food group was almost every day or occasionally, then 1 point was given; otherwise, no point was given. The DDS ranged from 0 to 9 points, with higher scores reflecting higher levels of dietary diversity.

# S05. The detailed parameters and processing of multimodal neuroimaging

# Structural 3D-T1-wighted imaging

# High-resolution 3D T1 scans were performed using the inversion recovery gradient recalled echo sequence with the following parameters: repetition time (TR)=7.3 ms, echo time (TE)=3.0 ms, inversion time=450 ms; flip angle=12°, field of view (FOV)=256 mm×256 mm, acquisition matrix=256×256, slice thickness=1.0 mm, slice number=176, and scan time=4 min 56 s.

# 7-delay pCASL

Scan parameters of the 7-delay pseudo-continuous arterial spin labeling (pCASL) sequence to obtain the CBF values were: TR = 9315.0 ms, TE = 11.2 ms, FOV = 220 mm × 220 mm, acquisition matrix = 512 × 512, 48 axial slices, thickness = 3.0 mm; the label durations of the seven labeling blocks were 0.361, 0.378, 0.402, 0.436, 0.491, 0.591, and 0.842 s, the post-labeling delays were 1.000, 1.361, 1.739, 2.141, 2.577, 3.067, and 3.658 s, and the scan time was 15 min 55 s. A junior radiologist with 5 years of experience in neuroradiology performed data processing using CereFlow software 1.0 (Anying Technology Beijing Co., Ltd., China), which was checked by a senior radiologist with 20 years of experience in neuroradiology. The following steps were performed: (1) importing automatically generated cerebral blood flow (CBF) and arterial transit time (ATT) images from the default vendor’s postprocessing pipeline; (2) co-registration of the M0 image (GE ASL’s PD image) with the anatomical T1-weighted image; the CBF/ATT images were also co-registered to the T1 image with the same transformation parameters; (3) normalization of the T1 images to the Montreal Neurological Institute template; (4) warping the CBF/ATT images into the Montreal Neurological Institute space using the forward transformation matrix derived from T1; and (5) regional CBF corrected by ATT (cCBF) were reconstructed.

**S06. The composition of malnourished diet and standard diet**

| Components | Malnourished diet  10%Fat+5%Protein | Standard diet  15%Fat+20%Protein |
| --- | --- | --- |
| Casein, g | 50 | 200 |
| Cystine, g | 0.75 | 3 |
| Starch, Corn, g | 527.25 | 346 |
| Dextrose, g | 0 | 250 |
| Sucrose, g | 254 | 4 |
| Maltodextrin, g | 75 | 45 |
| Solka Floc, g | 50 | 75 |
| Raftiline, g | 0 | 25 |
| Soybean Oil, g | 25 | 70 |
| Lard, g | 20 | 0 |
| S10026, g | 50 | 50 |
| Choline Bitartrate, g | 2 | 2 |
| V10001, g | 1 | 1 |
| Dye, Red, g | 0.025 | 0 |
| Dye, Blue, g | 0.025 | 0 |
| Total, g | 1055.05 | 1071 |
| Protein, cal% | 5 | 20 |
| Fat, cal% | 10 | 15 |
| Carbohydrate, cal% | 85 | 65 |
| Total Kcal/g | 3.9 | 3.9 |

**S07. The detailed process of behavioral testing in the mice study**

The open field test (OFT) was performed to evaluate anxiety-like behavior, as previous described [[17](#_ENREF_17)]. It consists of a square black Plexiglas box (length: 50 cm; width: 50 cm; height: 40 cm), with an outlined center area (25 cm × 25 cm). Each mouse was gently placed in the middle of the box and allowed to move freely within the box for 10 min. The moving distance and percentage of time spent in the center area during the test were recorded.

The tail suspension test (TST) and the forced swim test (FST) were performed on the next two days after the OFT to evaluate depression-like behavior, as previous described [[18](#_ENREF_18)]. In the TST, mice were suspended by the tails by using adhesive tape 1 cm from the tip of the tail in a 55 cm high white open box. Small plastic tubes were attached to tails to make sure mice could not climb. Each mouse was suspended by its tail for 6 min. The first 2 min was the adaptation period, and the last 4 min was the official tail suspension time. The immobility time in the last 4 min was observed and recorded with a video camera.

In the FST, each mouse was individually placed in an open transparent cylindrical container (diameter 10 cm, height 25 cm) filled with 16 cm depth of water at 23 ± 1 °C without the possibility of escaping and being forced to swim. The total amount of time each animal remained immobile for 6-minute sessions was recorded, in which only the last 4 minutes were analyzed. At the end of the test, was immediately dried and warmed using a dry paper towel before being returned to their home cage. The total immobile time was manually analyzed from video recordings.

The sucrose preference test (SPT) was performed following previously reported protocol [[19](#_ENREF_19)]. Before the SPT experiment, mice were habituated with two identical water bottles for 48h. Each mouse was water and food-deprived for 24 h and then provided with 1% water and sucrose solution. After 12 h, the bottles were weighted and the sucrose preference was calculated as [sucrose water intake/ (sucrose water intake + pure water intake)] × 100%.

Morris water maze test was performed to evaluate spatial learning and memory in the mice as before [[20](#_ENREF_20)]. The apparatus is a 1.6-m-diameter pool surrounded by four curtains. The pool was divided equally into four quadrants, with a hidden platform in one of the quadrants. Before the experiment, the pool was filled with water 1–2 cm above the platform. During the acquisition phase (Day 1–5), the mice were trained once a day and placed in four different quadrants to search for the platform for 1 min at a time. The duration of searching time is considered as latency time. Mice that have not found the platform in 1 min will be guided by the experimenter to stay on the platform for 30 s. In the probe test (Day 6), the platform was removed and a 60 s exploration experiment was performed to investigate the memory function of the mouse on the platform. the number and time in target quadrant were recorded and analyzed.

Y maze test was used to assess short-term spatial memory as previous description [[20](#_ENREF_20)]. The apparatus is a black maze with three arms (30 cm length × 60 cm width × 25 cm height) positioned 120° from each other. The YMT was performed by placing the mice in the center of Y‐maze and mice were allowed to freely explore the Y‐maze for 10 min. The entry sequence to each arm was recorded and analyzed to calculate spontaneous alternation percentage. Every entry was counted as valid only when all four limbs were placed in the arm.

**S08. The detailed protocols of western blot, immunofluorescence, and immunohistochemistry**

**Western blot**

The brain tissues (midbrain and corpus striatum) were homogenized in cold RIPA buffer (AR0102, BOSTER) containing a cocktail of PMSF (AR1179, BOSTER) and protein phosphatase inhibitor (AR1183, BOSTER). The homogenates were then centrifuged (12000 rpm, 25 min, 4 °C), and the supernatants were collected. The total protein concentrations of the samples were measured by a BCA Protein Assay Kit (P0012, Beyotime). Protein solutions from three mice in each group were denatured with 4 × loading buffer at 95 °C for 15 min and stored at −20 °C. Ten micrograms of total protein sample were separated by SDS-polyacrylamide gelelectrophoresis and transferred onto polyvinylidene fluoride (PVDF) membranes. Membranes were blocked in Tris-buffered saline/0.1% Tween buffer (TBST; 25 mM Tris–Cl, 125 mM NaCl, 0.1% Tween20) with 5% skim milk powder at room temperature for 90min and incubated with primary antibodies at 4 °C overnight (the details of primary antibodies were seen at Table S1). The samples were incubated with a Peroxidase AffiniPure™ Goat Anti-Rabbit IgG (H+L) secondary antibody (AB_2307391, 1:10000, Jackson ImmunoResearch laboratories, USA) for 1 h at room temperature. The protein expression levels were quantified using ImageJ (National Institutes of Health, Scion Corporation, USA), with each protein band being normalized to a loading control protein (β-actin) and presented as multiples of the relevant control. The protein bands were visualized using super-enhanced chemiluminescence detection reagents (Leica Clara Ltd., USA).

**Immunofluorescence (IF)**

One series of the 8-10 μm sections mentioned above was permeabilized, subjected to antigen retrieval, blocked, and then incubated with the primary antibodies (same as western blot) at 4 °C overnight. The corresponding Peroxidase AffiniPure™ Goat Anti-Rabbit IgG (H+L) secondary antibodies (AB_2307391, 1:200, Jackson ImmunoResearch laboratories, USA) were subsequently incubated at room temperature for 1h. Sections were stained with DAPI and sealed with an autofluorescence quencher. Images were captured using a confocal microscope (Zeiss) under a ×60oil lens. The image threshold was uniformly set using ImageJ v.6.4, and the number of labeled cells was counted manually with a double-blind procedure.

**Immunohistochemistry (IHC)**

The collected brains were immersed in 4% formalin, successively dehydrated in 75%, 85%, 95%, 100% ethanol gradient for 1 h each, embedded with paraffin. A Manual Rotary Microtome (Leica RM2235, Leica, Germany) was used to cut the brain tissues that including midbrain or striatum, into continuous sections at 4-μm thick sections. Three mice were selected randomly from each group, and one set of 4-μm-thick sections including midbrain or striatum was randomly selected from each mouse. Bake the slices in a 68 °C oven for 2-8 hours. The paraffin sections were deparaffinized in xylene (15min×2) and rehydrated in an ethanol gradient (100%, 90%, 80%, 70%). Then, antigen retrieval treatment was performed in citrate buffer in a microwave over medium heat for 1.5 mins and then cooled to room temperature. Next, these sections were washed three times with 0.01M PBS and permeabilized in PBS containing 0.3% Triton X-100/0.1% Tween-20 (PBST) for 15 min, incubated with 0.3% H2O2 for 10min to eliminate endogenous peroxidase activity. After washing with PBS buffer three times (3min×3), the sections were sealed by using goat serum (ZSGB Bio, Beijing, China) for 15 mins and incubated with primary antibodies against TH, ChAT, TPH, and NR2B (the details were seen at Table S1) at 37 °C for 1h. After incubation with HRP-conjugated secondary antibody solution (PV-6000, ZSGB-BiO) at room temperature for 30 mins, a 3,3′-diaminobenzidine (DAB) Kit (ZLI-9019, ZSGB-BiO) was used for staining. They were then counterstained with hematoxylin (15s), dehydrated with gradient alcohol series (75%, 85%, 95%, 100%), cleared with xylene, sealed with neutral gum and cover slipped. The evaluation of immunohistochemistry staining included the percentage of positive cells and localization of staining in midbrain or striatum.

**Table S1. Details of primary antibodies used in western blot, immunofluorescence, and** **immunohistochemistry**

| Antibodies | Molecular weight | Catalog number | Application and Dilution | Company |
| --- | --- | --- | --- | --- |
| Anti-TH antibody | 58 kDa/62 kDa | ab137869/AB152 | WB: 1:1000  IF-Fr: 1:200  IHC-P (striatum): 1:50000  IHC-P (midbrain): 1:100 | Abcam, UK/Merck, Germany |
| Anti-TPH antibody | 56 kDa | ab184505 | WB: 1:1000  IF-Fr: 1:200  IHC-P (striatum): 1:75  IHC-P (midbrain): 1:75 | Abcam, UK |
| Anti-NR2B antibody | 165kD | YT3152 | WB: 1:1000  IF-Fr: 1:200  IHC-P (striatum): 1:32000  IHC-P (midbrain): 1:32000 | ImmunoWay Biotechnology, USA |
| Anti-ChAT antibody | 82 kDa | ab181023 | WB: 1:1000  IF-Fr: 1:200  IHC-P (striatum): 1:700  IHC-P (midbrain): 1:700 | Abcam, UK |
| Anti-PSD95 antibody | 80 kDa | ET1602-20 | WB: 1:1000 | Huabio, China |
| Anti-β actin antibody |  | ab8227 | WB: 1:1000 | Abcam, UK |
| Anti-cAMP kinase catalytic subunit antibody | 46 kDa | ab76238 | WB: 1:20000 | Abcam, UK |
| Phospho-PKA C (Thr197) | 42 kDa | 5661T | WB 1:1000 | CST, USA |
| Phospho-CREB (Ser133) | 43 kDa | 9198T | WB 1:1000 | CST, USA |

Abbreviation: TH, tyrosine hydroxylase; TPH2, tryptophan hydroxylase; NR2B, N-methyl-d-aspartate receptor subunit 2B; ChAT, choline acetyltransferase; PSD95, postsynaptic density 95; cAMP, cyclic adenosine monophosphate; PKA, protein kinase A; CREB, cyclic adenosine monophosphate response element binding protein; CST, Cell Signaling Technology.

**S09. Quasi-targeted metabolomics analysis**

Brain samples (midbrain or corpus striatum) were homogenized with 100 μL of acetonitrile and sonicated for 20 min at 4 °C. This step was repeated three times and the samples were allowed to sit overnight at −20 °C. All samples were incubated on ice for 5 min and then centrifuged at 12000 rpm at 4 °C for 15 min. The supernatants (∼40 μL) were mixed with 20 μL of Na_2_CO_3_ solution (100 mM/L) and 20 μL of 2% benzoyl chloride acetonitrile solution and incubated at room temperature for 30 min. After the addition of 5 μL of the internal standard, the samples were centrifuged again at 12000 rpm for 15 min at 4 °C. The supernatants (20 μL) were mixed with 10 μL of acetonitrile in 0.1% formic acid and transferred to an autosampler vial to measure the neurotransmitter concentrations on a UHPLC–MS/MS (QTrap 6500 plus, AB Sciex Co., Ltd., USA). For other quality control/quality assurance, during the process, one blank was analyzed after four samples. The recoveries determined were 83.45–111.60% and relative standard deviations were below 8.74%.

Detection of the experimental samples using MRM was based on the Novogene in-house database. The final concentration (CF, nmol/L) equals the calculated concentration (Cc, nmol/L) multiplied by the dilution factor (Dil). The metabolite concentration (CM, nmol/kg) equals the final concentration (CF, nmol/L) multiplied by the final volume (VF, µL), and divided by the weight (Ms, mg) of the sample. Data files generated by UHPLC-MS/MS were processed with SCIEX OS (version 1.4) to integrate and correct the peaks. A total of 39 neurotransmitters compounds were identified in the samples, including Acetylcholine, 5-Hydroxyindoleacetic acid (5-HIAA), Serotonin (5-HT), 5-Hydroxytryptophan, L-Alanine, L(+)-Arginine, L-Asparagine, L-Aspartate, β-alanine, L-Cysteine, Dopamine (DA), 3,4-Dihydroxyphenylacetic acid (DOPAC), Epinephrine, 4-Aminobutyric acid (GABA), Glutathione, L-Glutamine, L-Glutamic acid, Glycine, Homovanillic acid, L-Histidine, Histamine, Kynurenine, 3,4-Dihydroxyphenylalanine, L-Leucine, L-Lysine, Methionine, Norepinephrine, Ornithine, L-Phenylalanine, Putrescine, Serine, Spermidine, Spermine, Threonine, L-Tryptophan, L-Tyrosine, Tyramine, L-Valine, and Melatonin. Metabolomics data analysis was then performed using MetaboAnalyst 4.0 (47).

**S10. Transcriptomic analysis of brain tissues**

1.1 Sample collection and preparation

(1) RNA extraction and detection

Plant samples were extracted by ethanol precipitation and CTAB-PBIOZOL. Animal samples were extracted by Trizol method. After successful extraction, RNA was dissolved by adding 50 µL of DEPC-treated water. Subsequently, total RNA was identified and quantified using a Qubit fluorescence quantifier and a Qsep400 high-throughput biofragment analyzer.

(2) mRNA library construction

1) By utilizing the structural characteristic that most eukaryotic mRNAs carry a polyA tail, mRNAs with polyA tails were enriched by Oligo(dT) magnetic beads;

2) The purified mRNAs were cleaved into small fragments with fragmentation buffer at a suitable temperature;

3) First-strand cDNAs were produced by reverse transcription using a random hexamer primer;

4) Second-strand cDNAs are synthesized (strand-specific library: dUTPs were used instead of dTTPs in the second-strand synthesis to incorporate dUTPs in the second-strand cDNAs, while the high-fidelity DNA polymerase used in this method could not amplify uracil-containing DNA templates, thus realizing the strand-specificity), while simultaneously performing end repair and dA-Tailing;

5) Sequencing adapter ligation was performed, followed by DNA magnetic bead purification and fragment selection after ligation was completed to yield a library with 250-350 bp insert fragments;

6) The ligated products were amplified by PCR and purified again using DNA magnetic beads, where the products were solubilized with nuclear-free water;

7) After the initial library was constructed, a Qubit fluorescence quantifier was used for concentration detection, followed by a Qsep400 high-throughput biofragment analyzer for fragment size detection;

8) Finally, the effective concentration of the library was accurately quantified using Q-PCR.

(3) Sequencing run

After passing the library check, the different libraries were sequenced in Illumina after pooling them according to the effective concentration and the target sequencing output data volume, yielding 150bp paired-end reads. The basic principle of sequencing is to synthesize and sequence at the same time. Four types of fluorescently labeled dNTPs, DNA polymerase, and junction primers were added to the sequencing flow cell for amplification. When extending the complementary strand of each sequencing cluster, each fluorescently labeled dNTP added emits corresponding fluorescence, and the sequencer captures the fluorescence signals, and converts the light signals into sequencing peaks through computer software, to obtain the sequence information of the fragment to be tested.

1.2 Data Analysis

(1) Data Quality Control Data

Quality control was performed using fastp to remove reads with adapters. Paired reads were removed under the following conditions: when the the number of N in any sequencing read exceeded 10% of the length of that read, and when any sequencing read contained low-quality bases (Q<=20) exceeding 50% of the length of that read. Subsequent analyses were based on clean reads.

(2) Sequence Alignment to the Reference Genome

The reference genome and its annotation files were downloaded from a specified website. HISAT was used to build an index, and clean reads were aligned to the reference genome.

(3) Quantification of Gene Expression

Levels Gene expression levels were quantified using featureCounts to calculate gene alignment statistics. Subsequently, FPKM (Fragments Per Kilobase Million) values for each gene were computed based on gene length. FPKM is currently the most used method for estimating gene expression levels.

(4) Differential Analysis DESeq2 was used for differential gene expression analysis between two groups, and Benjamini & Hochberg correction was applied to P-values. Corrected P-values and log2 fold change were used as thresholds for significant differential expression.

**Table S2. The baseline differences of MNA score between patients with NPS or its specific sub-type and those without**

| Outcome | Number | B | SE | Wald | P value | OR | 95% *CI* |
| --- | --- | --- | --- | --- | --- | --- | --- |
| General NPS | 285 | -0.251 | 0.065 | 14.85 | <0.001 | 0.778 | 0.685-0.884 |
| Delusion | 69 | 0.006 | 0.049 | 0.013 | 0.909 | 1.006 | 0.914-1.107 |
| Hallucination | 41 | 0.011 | 0.060 | 0.037 | 0.848 | 1.012 | 0.899-1.138 |
| Agitation | 91 | 0.010 | 0.044 | 0.053 | 0.818 | 1.010 | 0.927-1.101 |
| Depression | 146 | -0.123 | 0.041 | 8.997 | 0.003 | 0.844 | 0.815-0.958 |
| Anxiety | 164 | -0.082 | 0.039 | 4.346 | 0.037 | 0.921 | 0.853-0.995 |
| Euphoria | 26 | 0.037 | 0.074 | 0.244 | 0.621 | 1.037 | 0.998-1.039 |
| Apathy | 143 | -0.089 | 0.044 | 4.065 | 0.044 | 0.914 | 0.838-0.998 |
| Disinhibition | 49 | -0.016 | 0.055 | 0.082 | 0.775 | 0.984 | 0.884-1.096 |
| Irritability | 114 | -0.037 | 0.041 | 0.807 | 0.369 | 0.964 | 0.890-1.044 |
| Aberrant motor activity | 41 | 0.006 | 0.059 | 0.011 | 0.918 | 1.006 | 0.896-1.130 |
| Sleep/nighttime behavior | 101 | -0.065 | 0.042 | 2.394 | 0.122 | 0.937 | 0.864-1.017 |
| Appetite/eating disturbances | 51 | -0.215 | 0.053 | 16.653 | <0.001 | 0.806 | 0.727-0.894 |

The multivariate logistics regression models were developed to evaluate the baseline differences of MNA score between patients with NPS or its specific sub-type and those without after adjusting for age, sex, BMI, MMSE score and CBI score.

NPS, neuropsychiatric symptoms; BMI, body mass index; MNA, Mini-Nutritional Assessment; MMSE, Mini-mental State Examination; CBI, Caregiver Burden Inventory.

**Table S3. The baseline clinical profiles of patients on the AD continuum with follow-up and those lost to follow-up**

| Variables | Overall (n=374) | Followed up  (n=136) | Dropped out  (n=238) | t/χ2/Z | *P* value |
| --- | --- | --- | --- | --- | --- |
| Age [years, M±SD] | 66.54±8.32 | 67.65±8.02 | 65.90±8.43 | -1.966 | 0.050 |
| Sex (female, %) | 226.00 (60.43) | 79.00 (58.09) | 147.00 (61.76) | 0.489 | 0.484 |
| BMI (kg/m2, M±SD) | 23.91±3.25 | 23.96±3.16 | 23.88±3.31 | -0.209 | 0.835 |
| WHR (M±SD) | 0.88±0.08 | 0.87±0.09 | 0.88±0.07 | 0.550 | 0.583 |
| Education [years, median (IQR)] | 11.00 (9.00, 13.00) | 12.00 (9.00, 13.00) | 11.00 (9.00, 13.00) | -0.315 | 0.753 |
| Marital status (married, %) | 327.00 (87.43) | 121.00 (88.97) | 206.00 (86.55) | 0.460 | 0.498 |
| *APOE*ε4 carrier (yes, %) | 115.00 (30.75) | 42.00 (30.88) | 73.00 (30.67) | 1.688 | 0.194 |
| Hypertension (yes, %) | 166.00 (44.39) | 59.00 (43.38) | 107.00 (44.96) | 0.087 | 0.768 |
| Diabetes mellitus (yes, %) | 64.00 (17.11) | 21.00 (15.44) | 43.00 (18.07) | 0.421 | 0.517 |
| Cerebrovascular disease (yes, %) | 67.00 (17.91) | 23.00 (16.91) | 44.00 (18.49) | 0.146 | 0.702 |
| Coronary heart disease (yes, %) | 74.00 (19.79) | 24.00 (17.65) | 50.00 (21.01) | 0.616 | 0.432 |
| Dyslipidemia (yes, %) | 161.00 (43.05) | 59.00 (43.38) | 102.00 (42.86) | 0.004 | 0.948 |
| Smoking (yes, %) | 81.00 (21.66) | 32.00 (23.53) | 49.00 (20.59) | 0.441 | 0.507 |
| Alcohol consumption (yes, %) | 110.00 (29.41) | 44.00 (32.36) | 66.00 (27.73) | 0.890 | 0.345 |
| MNA [score, medians (IQRs)] | 24.00 (21.50, 26.00) | 24.25 (22.00, 26.38) | 24.00 (21.50, 26.00) | -1.240 | 0.215 |
| MMSE [score, medians (IQRs)] | 24.00 (17.75, 27.00) | 25.00 (19.00, 27.00) | 24.00 (16.75, 27.00) | -2.123 | 0.034 |
| MoCA [score, medians (IQRs)] | 19.00 (11.00, 22.00) | 19.00 (13.00, 23.00) | 18.00 (11.00, 22.00) | -1.611 | 0.107 |
| CBI [score, medians (IQRs)] | 5.00 (0.00, 23.00) | 5.50 (0.00, 24.75) | 4.00 (0.00, 23.00) | -0.115 | 0.909 |
| NPI [score, medians (IQRs)] | 5.00 (0.00, 13.00) | 5.00 (1.00, 13.00) | 4.00 (0.00, 13.00) | -1.005 | 0.315 |
| HAMD [score, medians (IQRs)] | 6.00 (3.00, 10.00) | 5.50 (2.00, 10.00) | 6.00 (3.00, 10.00) | -1.007 | 0.314 |
| HAMA [score, medians (IQRs)] | 5.00 (3.00, 9.00) | 4.00 (2.75, 8.00) | 5.00 (3.00, 9.00) | -0.716 | 0.474 |
| General NPS (yes, %) | 285.00 (76.20) | 110.00 (80.88) | 175.00 (73.53) | 2.580 | 0.108 |
| Delusion (yes, %) | 69.00 (18.45) | 30.00 (22.06) | 39.00 (16.39) | 1.851 | 0.174 |
| Hallucination (yes, %) | 41.00 (10.96) | 20.00 (14.71) | 21.00 (8.82) | 3.068 | 0.080 |
| Agitation (yes, %) | 91.00 (24.33) | 40.00 (29.41) | 51.00 (21.42) | 2.996 | 0.083 |
| Depression (yes, %) | 146.00 (39.04) | 61.00 (44.85) | 85.00 (35.71) | 3.037 | 0.081 |
| Anxiety (yes, %) | 164.00 (43.85) | 62.00 (45.59) | 102.00 (42.86) | 0.262 | 0.609 |
| Euphoria (yes, %) | 26.00 (6.95) | 12.00 (8.82) | 14.00 (5.88) | 1.157 | 0.282 |
| Apathy (yes, %) | 143.00 (38.24) | 55.00 (40.44) | 88.00 (36.97) | 0.440 | 0.507 |
| Disinhibition (yes, %) | 49.00 (13.10) | 16.00 (11.76) | 33.00 (13.87) | 0.336 | 0.562 |
| Irritability (yes, %) | 114.00 (30.48) | 44.00 (32.35) | 70.00 (29.41) | 0.353 | 0.552 |
| Aberrant motor activity (yes, %) | 41.00 (10.96) | 13.00 (9.56) | 28.00 (11.76) | 0.431 | 0.511 |
| Sleep/nighttime behavior (yes, %) | 101.00 (27.01) | 41.00 (30.15) | 60.00 (25.21) | 1.070 | 0.301 |
| Appetite/eating disturbance (yes, %) | 51.00 (13.63) | 20.00 (14.71) | 31.00 (13.03) | 0.208 | 0.649 |

Data are shown as the mean ± standard deviation, medians with interquartile ranges (IQRs) or n (%).

AD, Alzheimer’s disease; NPS, neuropsychiatric symptoms; BMI, body mass index; WHR, waist to hip ratio; *APOEε4*, apolipoprotein E type epsilon 4; MNA, Mini-Nutritional Assessment; MMSE, Mini-mental State Examination; MoCA, Montreal Cognitive Assessment; NPI, Neuropsychiatric Inventory; HAMD, Hamilton Depression Scale; HAMA, Hamilton Anxiety Scale; CBI, Caregiver Burden Inventory.

**S11.** **The nutritional status of AD mice, ranging from young to old, before malnutrition treatment**


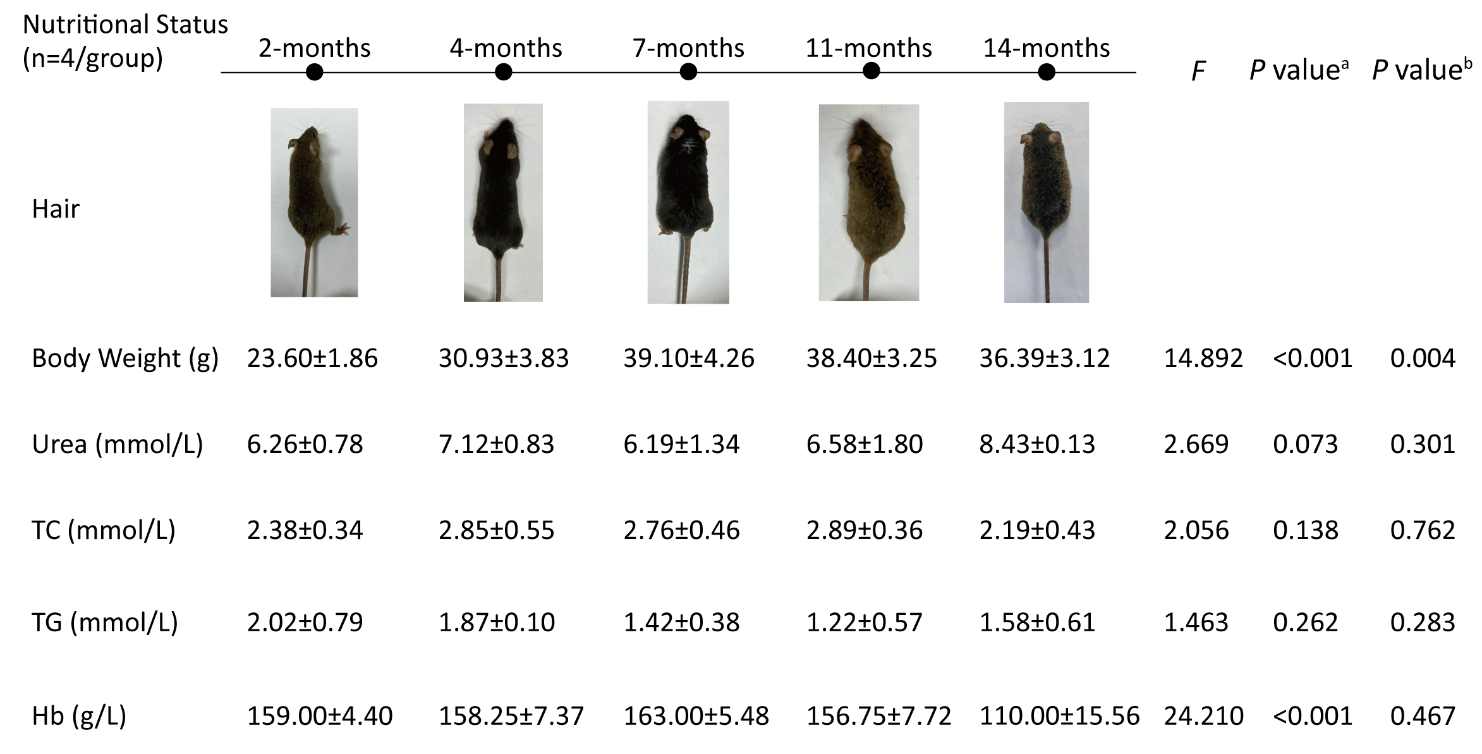


Abbreviations: AD, Alzheimer’s disease; TC, total cholesterol; TG, triglyceride; Hb, hemoglobin.

**Table S4. The nutritional biomarkers among the four groups at baseline**

| Variables | WTSD | WTMD | ADSD | ADMD | t/Z | *P* value ^a^ | *P* value ^b^ |
| --- | --- | --- | --- | --- | --- | --- | --- |
| Hb | 148.00±6.89 | 152.00±8.35 | 150.13±7.08 | 154.38±8.67 | -1.074 | 0.301 | 0.422 |
| ALB | 27.41±0.92 | 28.36±1.44 | 28.73±1.18 | 28.64±1.96 | 0.108 | 0.915 | 0.125 |
| BUN | 9.50±0.80 | 9.07±0.89 | 9.08±2.05 | 8.95±0.95 | 0.160 | 0.876 | 0.544 |
| GLU | 13.30±3.11 | 11.49±4.21 | 10.81±4.74 | 14.35±3.21 | -1.752 | 0.102 | 0.898 |
| TC | 1.55±0.29 | 1.69±0.33 | 1.72±0.67 | 1.85±0.28 | -0.533 | 0.602 | 0.275 |
| TG | 0.73±0.31 | 0.53±0.15 | 0.84±0.15 | 0.76±0.10 | 1.226 | 0.240 | 0.021 |
| VitB_12_ | 9.29±7.51 | 8.38±5.28 | 17.72±4.07 | 17.03±4.04 | 0.342 | 0.737 | <0.001 |
| VitD_3_ | 221.83±47.12 | 187.69±41.02 | 160.66±71.80 | 138.87±128.01 | 0.420 | 0.681 | 0.057 |

^a^ the p value between ADSD and ADMD; ^b^ the p value between WT and AD.

Abbreviations: Hb, hemoglobin; ALB, albumin; BUN, blood urea nitrogen; GLU, glucose; TC, total cholesterol; TG, triglyceride; VitB_12_, vitamin B12; VitD_3_, vitamin D3.

**Figure S1.** **Structural T2-weighted imaging of the four groups after two-month dietary intervention**


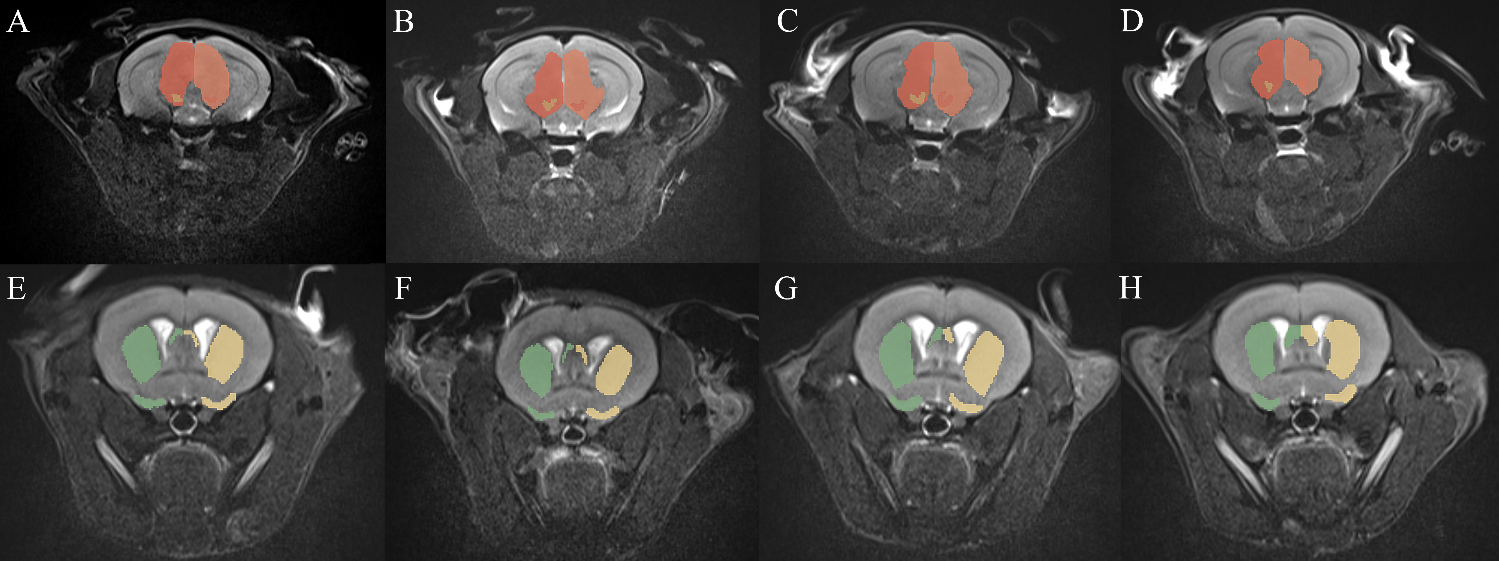


The volumes of left midbrain among four groups were 22.35±1.27, 22.59±0.42, 23.21±1.81, and 19.83±3.02, and the volumes of right midbrain among four groups were 22.77±0.47, 21.62±0.62, 22.55±2.08, and 19.38±2.27 (A-D). The volumes of left striatum among four groups were 13.34±2.04, 12.66±0.33, 13.70±0.01, and 11.73±0.38, and the volumes of right striatum among four groups were 12.87±1.07, 11.99±0.33, 14.43±0.65, and 12.45±0.51 (E-H).

**Table S5. The Q values of all enriched metabolic pathways of differential neurotransmitters in both midbrain and striatum based on the KEGG database**

| Pathway | Description | Compounds | P value | Q value |
| --- | --- | --- | --- | --- |
| mmu04974 | Protein digestion and absorption | 21 | 3.8E-35 | 3.96E-33 |
| mmu00970 | Aminoacyl-tRNA biosynthesis | 17 | 3.25E-25 | 1.69E-23 |
| mmu05230 | Central carbon metabolism in cancer | 15 | 7.42E-24 | 2.57E-22 |
| mmu01230 | Biosynthesis of amino acids | 18 | 1.74E-19 | 4.54E-18 |
| mmu00470 | D-Amino acid metabolism | 14 | 2.21E-17 | 4.6E-16 |
| mmu02010 | ABC transporters | 17 | 2.54E-17 | 4.4E-16 |
| mmu04080 | Neuroactive ligand-receptor interaction | 13 | 2.57E-17 | 3.81E-16 |
| mmu04721 | Synaptic vesicle cycle | 8 | 3.11E-15 | 4.04E-14 |
| mmu04978 | Mineral absorption | 10 | 4.18E-15 | 4.83E-14 |
| mmu00480 | Glutathione metabolism | 8 | 2.66E-10 | 2.77E-09 |
| mmu04024 | cAMP signaling pathway | 6 | 2.46E-08 | 2.33E-07 |
| mmu00250 | Alanine, aspartate and glutamate metabolism | 6 | 5.15E-08 | 4.46E-07 |
| mmu05030 | Cocaine addiction | 4 | 1.12E-07 | 8.98E-07 |
| mmu00410 | beta-Alanine metabolism | 6 | 1.21E-07 | 9E-07 |
| mmu05031 | Amphetamine addiction | 4 | 4E-07 | 2.77E-06 |
| mmu01240 | Biosynthesis of cofactors | 13 | 5.24E-07 | 3.41E-06 |
| mmu01210 | 2-Oxocarboxylic acid metabolism | 9 | 5.85E-07 | 3.58E-06 |
| mmu05034 | Alcoholism | 4 | 6.62E-07 | 3.83E-06 |
| mmu00220 | Arginine biosynthesis | 5 | 6.94E-07 | 3.8E-06 |
| mmu00330 | Arginine and proline metabolism | 7 | 7.74E-07 | 4.03E-06 |
| mmu04540 | Gap junction | 4 | 1.03E-06 | 5.12E-06 |
| mmu00260 | Glycine, serine and threonine metabolism | 6 | 1.5E-06 | 7.08E-06 |
| mmu04728 | Dopaminergic synapse | 4 | 1.54E-06 | 6.98E-06 |
| mmu00350 | Tyrosine metabolism | 7 | 1.8E-06 | 7.81E-06 |
| mmu00770 | Pantothenate and CoA biosynthesis | 5 | 2.82E-06 | 1.17E-05 |
| mmu04742 | Taste transduction | 5 | 3.95E-06 | 1.58E-05 |
| mmu05033 | Nicotine addiction | 3 | 1.55E-05 | 5.95E-05 |
| mmu01100 | Metabolic pathways | 34 | 3.27E-05 | 0.000122 |
| mmu04727 | GABAergic synapse | 3 | 3.67E-05 | 0.000132 |
| mmu04976 | Bile secretion | 6 | 9.14E-05 | 0.000317 |
| mmu00270 | Cysteine and methionine metabolism | 5 | 0.000155 | 0.000521 |
| mmu01200 | Carbon metabolism | 6 | 0.000224 | 0.000728 |
| mmu04150 | mTOR signaling pathway | 2 | 0.00036 | 0.001134 |
| mmu00340 | Histidine metabolism | 4 | 0.000457 | 0.001398 |
| mmu00290 | Valine, leucine and isoleucine biosynthesis | 3 | 0.000717 | 0.002131 |
| mmu00430 | Taurine and hypotaurine metabolism | 3 | 0.000815 | 0.002355 |
| mmu04216 | Ferroptosis | 3 | 0.001433 | 0.004027 |
| mmu05207 | Chemical carcinogenesis - receptor activation | 3 | 0.001433 | 0.003921 |
| mmu00630 | Glyoxylate and dicarboxylate metabolism | 4 | 0.00148 | 0.003947 |
| mmu04724 | Glutamatergic synapse | 2 | 0.001647 | 0.004282 |
| mmu05032 | Morphine addiction | 2 | 0.001647 | 0.004177 |
| mmu00730 | Thiamine metabolism | 3 | 0.001743 | 0.004317 |
| mmu05022 | Pathways of neurodegeneration - multiple diseases | 3 | 0.001913 | 0.004628 |
| mmu04713 | Circadian entrainment | 2 | 0.002107 | 0.00498 |
| mmu00400 | Phenylalanine, tyrosine and tryptophan biosynthesis | 3 | 0.002484 | 0.005741 |
| mmu04261 | Adrenergic signaling in cardiomyocytes | 2 | 0.002621 | 0.005925 |
| mmu04122 | Sulfur relay system | 2 | 0.003187 | 0.007052 |
| mmu04917 | Prolactin signaling pathway | 2 | 0.003187 | 0.006905 |
| mmu00380 | Tryptophan metabolism | 4 | 0.003849 | 0.00817 |
| mmu04726 | Serotonergic synapse | 3 | 0.004196 | 0.008727 |
| mmu04923 | Regulation of lipolysis in adipocytes | 2 | 0.005195 | 0.010594 |
| mmu04971 | Gastric acid secretion | 2 | 0.005195 | 0.010391 |
| mmu05014 | Amyotrophic lateral sclerosis | 2 | 0.005195 | 0.010194 |
| mmu04924 | Renin secretion | 2 | 0.00765 | 0.014733 |
| mmu04970 | Salivary secretion | 2 | 0.00765 | 0.014465 |
| mmu04964 | Proximal tubule bicarbonate reclamation | 2 | 0.00765 | 0.014207 |
| mmu00910 | Nitrogen metabolism | 2 | 0.009524 | 0.017377 |
| mmu04723 | Retrograde endocannabinoid signaling | 2 | 0.009524 | 0.017078 |
| mmu05012 | Parkinson disease | 2 | 0.017487 | 0.030824 |
| mmu00920 | Sulfur metabolism | 2 | 0.027449 | 0.047578 |
| mmu04750 | Inflammatory mediator regulation of TRP channels | 2 | 0.03063 | 0.052221 |
| mmu04068 | FoxO signaling pathway | 1 | 0.038884 | 0.065225 |
| mmu04810 | Regulation of actin cytoskeleton | 1 | 0.038884 | 0.06419 |
| mmu05310 | Asthma | 1 | 0.038884 | 0.063187 |
| mmu00280 | Valine, leucine and isoleucine degradation | 2 | 0.042831 | 0.068529 |
| mmu04916 | Melanogenesis | 1 | 0.046483 | 0.073245 |
| mmu05142 | Chagas disease | 1 | 0.046483 | 0.072152 |
| mmu05016 | Huntington disease | 1 | 0.046483 | 0.071091 |
| mmu00650 | Butanoate metabolism | 2 | 0.052479 | 0.079098 |
| mmu04720 | Long-term potentiation | 1 | 0.054023 | 0.080263 |
| mmu05017 | Spinocerebellar ataxia | 1 | 0.054023 | 0.079132 |
| mmu00360 | Phenylalanine metabolism | 2 | 0.056537 | 0.081665 |
| mmu04915 | Estrogen signaling pathway | 1 | 0.061505 | 0.087624 |
| mmu05143 | African trypanosomiasis | 1 | 0.061505 | 0.08644 |
| mmu04929 | GnRH secretion | 1 | 0.06893 | 0.095583 |
| mmu04730 | Long-term depression | 1 | 0.06893 | 0.094325 |
| mmu00760 | Nicotinate and nicotinamide metabolism | 2 | 0.069341 | 0.093656 |
| mmu00564 | Glycerophospholipid metabolism | 2 | 0.071561 | 0.095415 |
| mmu00310 | Lysine degradation | 2 | 0.071561 | 0.094207 |
| mmu04072 | Phospholipase D signaling pathway | 1 | 0.083609 | 0.108692 |
| mmu04664 | Fc epsilon RI signaling pathway | 1 | 0.083609 | 0.10735 |
| mmu00240 | Pyrimidine metabolism | 2 | 0.090123 | 0.114302 |
| mmu04911 | Insulin secretion | 1 | 0.090864 | 0.113853 |
| mmu04725 | Cholinergic synapse | 1 | 0.090864 | 0.112498 |
| mmu05146 | Amoebiasis | 1 | 0.098063 | 0.119983 |
| mmu00860 | Porphyrin metabolism | 3 | 0.110039 | 0.133071 |
| mmu04071 | Sphingolipid signaling pathway | 1 | 0.112295 | 0.134238 |
| mmu04972 | Pancreatic secretion | 1 | 0.112295 | 0.132712 |
| mmu04270 | Vascular smooth muscle contraction | 1 | 0.119329 | 0.139441 |
| mmu04918 | Thyroid hormone synthesis | 1 | 0.153695 | 0.177604 |
| mmu04714 | Thermogenesis | 1 | 0.167074 | 0.190942 |
| mmu00230 | Purine metabolism | 2 | 0.189159 | 0.215888 |
| mmu00600 | Sphingolipid metabolism | 1 | 0.193218 | 0.218149 |
| mmu00450 | Selenocompound metabolism | 1 | 0.193218 | 0.215828 |
| mmu00750 | Vitamin B6 metabolism | 1 | 0.205989 | 0.227672 |
| mmu00780 | Biotin metabolism | 1 | 0.205989 | 0.2253 |
| mmu05415 | Diabetic cardiomyopathy | 1 | 0.266955 | 0.288972 |
| mmu00640 | Propanoate metabolism | 1 | 0.278591 | 0.29849 |
| mmu00120 | Primary bile acid biosynthesis | 1 | 0.312431 | 0.331366 |
| mmu00440 | Phosphonate and phosphonate metabolism | 1 | 0.360317 | 0.378333 |
| mmu05208 | Chemical carcinogenesis - reactive oxygen species | 1 | 0.365434 | 0.379906 |
| mmu01232 | Nucleotide metabolism | 1 | 0.37051 | 0.381407 |
| mmu00130 | Ubiquinone and other terpenoid-quinone biosynthesis | 1 | 0.43302 | 0.441428 |
| mmu00524 | Neomycin, kanamycin and gentamicin biosynthesis | 1 | 0.476959 | 0.481545 |

**Table S6.** **All the** **differentially expressed genes in midbrain and striatum of mice between ADMD group and ADSD group**

| Gene ID | Gene name | *P* value | *P*_FDR_ value | Regulated | Chromosome |
| --- | --- | --- | --- | --- | --- |
| **Striatum** |  |  |  |  |  |
| ENSMUSG00000035202 | Lars2 | 4.98537E-10 | 1.21663E-05 | up | 9 |
| ENSMUSG00000105153 | Gm3143 | 4.29346E-09 | 3.49259E-05 | down | 3 |
| ENSMUSG00000117278 | Gm36684 | 3.16385E-08 | 0.000154421 | down | 17 |
| ENSMUSG00000021250 | c-Fos | 8.43701E-08 | 0.000343161 | down | 12 |
| ENSMUSG00000071984 | Fndc1 | 1.20988E-05 | 0.042179932 | down | 17 |
| **Midbrain** |  |  |  |  |  |
| ENSMUSG00000057666 | Gapdh | 1.26429E-13 | 1.13907E-09 | down | 6 |
| ENSMUSG00000121395 | ENSMUSG00000121395 | 1.27015E-13 | 1.13907E-09 | down | 17 |
| ENSMUSG00000074768 | Bhmt | 3.44483E-09 | 1.54466E-05 | up | 13 |
| ENSMUSG00000021250 | c-Fos | 9.99969E-09 | 3.5819E-05 | down | 12 |
| ENSMUSG00000058207 | Serpina3k | 1.19823E-08 | 3.5819E-05 | up | 12 |
| ENSMUSG00000071984 | Fndc1 | 2.59427E-08 | 5.81636E-05 | down | 17 |
| ENSMUSG00000117278 | Gm36684 | 2.37523E-08 | 5.81636E-05 | down | 17 |
| ENSMUSG00000079429 | Mroh2a | 5.15853E-08 | 0.000102804 | up | 1 |
| ENSMUSG00000027513 | Pck1 | 6.72473E-08 | 0.000120615 | up | 2 |
| ENSMUSG00000029445 | Hpd | 7.97741E-08 | 0.000130075 | up | 5 |
| ENSMUSG00000052854 | Nrk | 2.17117E-07 | 0.000278158 | up | X |
| ENSMUSG00000105222 | Gm42205 | 2.84209E-07 | 0.000339839 | down | 3 |
| ENSMUSG00000014198 | Zfp385c | 3.3804E-07 | 0.000378943 | up | 11 |
| ENSMUSG00000035540 | Gc | 4.66936E-07 | 0.000492645 | up | 5 |
| ENSMUSG00000025479 | Cyp2e1 | 1.11481E-06 | 0.001052385 | up | 7 |
| ENSMUSG00000096255 | Dynlt1b | 1.08133E-06 | 0.001052385 | down | 17 |
| ENSMUSG00000071178 | Serpina1b | 1.67831E-06 | 0.001433437 | up | 12 |
| ENSMUSG00000059908 | Mug1 | 1.79981E-06 | 0.001467333 | up | 6 |
| ENSMUSG00000022868 | Ahsg | 2.47109E-06 | 0.001846729 | up | 16 |
| ENSMUSG00000066154 | Mup3 | 2.58207E-06 | 0.001852477 | up | 4 |
| ENSMUSG00000090461 | Il22b | 4.21502E-06 | 0.002800022 | up | 10 |
| ENSMUSG00000097248 | Gm2694 | 5.26349E-06 | 0.003314373 | up | 8 |
| ENSMUSG00000005681 | Apoa2 | 5.82478E-06 | 0.003416455 | up | 1 |
| ENSMUSG00000105153 | Gm3143 | 5.90489E-06 | 0.003416455 | down | 3 |
| ENSMUSG00000064201 | Krt2 | 6.77178E-06 | 0.003686959 | down | 15 |
| ENSMUSG00000020428 | Gabra6 | 7.58241E-06 | 0.003999943 | up | 11 |
| ENSMUSG00000066366 | Serpina1a | 7.84431E-06 | 0.004019874 | up | 12 |
| ENSMUSG00000033450 | Tagap | 9.38218E-06 | 0.004428387 | down | 17 |
| ENSMUSG00000004630 | Pcp2 | 9.7852E-06 | 0.00446864 | up | 8 |
| ENSMUSG00000032021 | Crtam | 1.11711E-05 | 0.0047706 | up | 9 |
| ENSMUSG00000052837 | Junb | 1.6364E-05 | 0.006380527 | down | 8 |
| ENSMUSG00000072849 | Serpina1e | 2.35101E-05 | 0.008433528 | up | 12 |
| ENSMUSG00000032083 | Apoa1 | 2.68444E-05 | 0.009259262 | up | 9 |
| ENSMUSG00000030359 | Pzp | 3.04251E-05 | 0.009912698 | up | 6 |
| ENSMUSG00000025991 | Cps1 | 3.62021E-05 | 0.010472922 | up | 1 |
| ENSMUSG00000042096 | Dao | 3.79327E-05 | 0.010630628 | up | 5 |
| ENSMUSG00000057400 | Ces1c | 4.09996E-05 | 0.010768706 | up | 8 |
| ENSMUSG00000029135 | Fosl2 | 4.82917E-05 | 0.011865214 | down | 5 |
| ENSMUSG00000026094 | Stk17b | 5.16176E-05 | 0.012181754 | up | 1 |
| ENSMUSG00000033860 | Fgg | 5.33121E-05 | 0.012418263 | up | 3 |
| ENSMUSG00000001670 | Tat | 6.51136E-05 | 0.013903314 | up | 8 |
| ENSMUSG00000026065 | Slc9a4 | 6.47072E-05 | 0.013903314 | down | 1 |
| ENSMUSG00000027843 | Ptpn22 | 6.94408E-05 | 0.014315977 | up | 3 |
| ENSMUSG00000066072 | Cyp4a10 | 7.6195E-05 | 0.015184813 | up | 4 |
| ENSMUSG00000068086 | Cyp2d9 | 8.24222E-05 | 0.015882059 | up | 15 |
| ENSMUSG00000085845 | Gm13944 | 8.52225E-05 | 0.016090006 | up | 2 |
| ENSMUSG00000028186 | Uox | 0.000108527 | 0.019272762 | up | 3 |
| ENSMUSG00000040380 | Cbln3 | 0.000111603 | 0.019434123 | up | 14 |
| ENSMUSG00000073542 | Cep76 | 0.000111232 | 0.019434123 | up | 18 |
| ENSMUSG00000087040 | Gm14033 | 0.000117869 | 0.020134279 | up | 2 |
| ENSMUSG00000020651 | Slc26a4 | 0.000130953 | 0.021160046 | down | 12 |
| ENSMUSG00000043144 | Aqp6 | 0.000133735 | 0.021240297 | up | 15 |
| ENSMUSG00000044244 | Il20rb | 0.000148809 | 0.022619026 | up | 9 |
| ENSMUSG00000089815 | Gm5083 | 0.000155161 | 0.023191398 | up | 13 |
| ENSMUSG00000071177 | Serpina1d | 0.000160439 | 0.023782172 | up | 12 |
| ENSMUSG00000098659 | 1110015O18Rik | 0.000163304 | 0.024008302 | up | 3 |
| ENSMUSG00000041261 | Car8 | 0.000182275 | 0.026365206 | up | 4 |
| ENSMUSG00000021922 | Itih4 | 0.000205 | 0.029181579 | up | 14 |
| ENSMUSG00000028438 | Kif24 | 0.000214819 | 0.029682606 | down | 4 |
| ENSMUSG00000037868 | Egr2 | 0.000214217 | 0.029682606 | down | 10 |
| ENSMUSG00000052087 | Rgs14 | 0.000233393 | 0.031474704 | down | 13 |
| ENSMUSG00000022875 | Kng1 | 0.000246504 | 0.032688359 | up | 16 |
| ENSMUSG00000029061 | Mmp23 | 0.000286633 | 0.036893209 | up | 4 |
| ENSMUSG00000002930 | Ppp1r17 | 0.000294302 | 0.036913351 | up | 6 |
| ENSMUSG00000085609 | 1700016P03Rik | 0.00033201 | 0.039747237 | down | 11 |
| ENSMUSG00000040017 | Saa4 | 0.000352187 | 0.041704366 | up | 7 |
| ENSMUSG00000002020 | Ltbp2 | 0.0003865 | 0.045308939 | up | 12 |

**Table S7. The Q values of all enriched metabolic pathways of *c-Fos* in both midbrain and striatum based on the KEGG database**

| KEGG map | Description | *P* value | *P*_FDR_ value |
| --- | --- | --- | --- |
| mmu05031 | Amphetamine addiction | 0.0152 | 0.0456 |
| mmu05140 | Leishmaniasis | 0.01542 | 0.0456 |
| mmu04917 | Prolactin signaling pathway | 0.0163 | 0.05543 |
| mmu05133 | Pertussis | 0.01695 | 0.06396 |
| mmu04662 | B cell receptor signaling pathway | 0.01827 | 0.0456 |
| mmu05323 | Rheumatoid arthritis | 0.01915 | 0.0456 |
| mmu05235 | PD-L1 expression and PD-1 checkpoint pathway in cancer | 0.01936 | 0.0456 |
| mmu05210 | Colorectal cancer | 0.01936 | 0.0456 |
| mmu04658 | Th1 and Th2 cell differentiation | 0.01936 | 0.0456 |
| mmu01522 | Endocrine resistance | 0.02046 | 0.0456 |
| mmu04657 | IL-17 signaling pathway | 0.02046 | 0.0456 |
| mmu05231 | Choline metabolism in cancer | 0.02155 | 0.0456 |
| mmu04713 | Circadian entrainment | 0.02155 | 0.0456 |
| mmu04620 | Toll-like receptor signaling pathway | 0.02199 | 0.0456 |
| mmu05142 | Chagas disease | 0.02265 | 0.0456 |
| mmu04660 | T cell receptor signaling pathway | 0.02265 | 0.0456 |
| mmu04659 | Th17 cell differentiation | 0.02308 | 0.0456 |
| mmu04928 | Parathyroid hormone synthesis, secretion and action | 0.02374 | 0.0456 |
| mmu04725 | Cholinergic synapse | 0.02461 | 0.04622 |
| mmu04668 | TNF signaling pathway | 0.02527 | 0.05543 |
| mmu04935 | Growth hormone synthesis, secretion and action | 0.0257 | 0.05543 |
| mmu04380 | Osteoclast differentiation | 0.0281 | 0.05543 |
| mmu04926 | Relaxin signaling pathway | 0.02832 | 0.05543 |
| mmu04915 | Estrogen signaling pathway | 0.02941 | 0.05645 |
| mmu05135 | Yersinia infection | 0.02963 | 0.05645 |
| mmu04728 | Dopaminergic synapse | 0.02963 | 0.05645 |
| mmu04210 | Apoptosis | 0.02985 | 0.05645 |
| mmu05162 | Measles | 0.03202 | 0.0456 |
| mmu05224 | Breast cancer | 0.03224 | 0.0456 |
| mmu05418 | Fluid shear stress and atherosclerosis | 0.03224 | 0.0456 |
| mmu04921 | Oxytocin signaling pathway | 0.03355 | 0.0456 |
| mmu04932 | Non-alcoholic fatty liver disease | 0.0342 | 0.0456 |
| mmu05161 | Hepatitis B | 0.03572 | 0.0456 |
| mmu05417 | Lipid and atherosclerosis | 0.04719 | 0.0456 |
| mmu05208 | Chemical carcinogenesis - reactive oxygen species | 0.04849 | 0.0456 |
| mmu04024 | cAMP signaling pathway | 0.04892 | 0.0456 |
| mmu05167 | Kaposi sarcoma-associated herpesvirus infection | 0.04892 | 0.0456 |
| mmu05207 | Chemical carcinogenesis - receptor activation | 0.04914 | 0.0456 |
| mmu05170 | Human immunodeficiency virus 1 infection | 0.05237 | 0.0456 |
| mmu05171 | Coronavirus disease - COVID-19 | 0.05409 | 0.0456 |
| mmu05166 | Human T-cell leukemia virus 1 infection | 0.05452 | 0.0456 |
| mmu05132 | Salmonella infection | 0.05516 | 0.0456 |
| mmu04010 | MAPK signaling pathway | 0.06396 | 0.0456 |

**Table S8. The Q values of all enriched pathways of *c-Fos* in both midbrain and striatum based on the GO database**

| GO term | GO | Description | *P* value | *P*_FDR_ value |
| --- | --- | --- | --- | --- |
| Molecular function | GO:0000979 | RNA polymerase II core promoter sequence-specific DNA binding | 0.003235 | 0.01498 |
| Molecular function | GO:0070412 | R-SMAD binding | 0.003516 | 0.01498 |
| Molecular function | GO:0001046 | core promoter sequence-specific DNA binding | 0.007163 | 0.01552 |
| Molecular function | GO:0046332 | SMAD binding | 0.011917 | 0.022132 |
| Molecular function | GO:0001221 | transcription coregulator binding | 0.0186 | 0.026866 |
| Biological process | GO:0035994 | response to muscle stretch | 0.002899 | 0.039236 |
| Biological process | GO:0071276 | cellular response to cadmium ion | 0.003589 | 0.039236 |
| Biological process | GO:0045672 | positive regulation of osteoclast differentiation | 0.004829 | 0.039236 |
| Biological process | GO:0046686 | response to cadmium ion | 0.005242 | 0.039236 |
| Biological process | GO:1902895 | positive regulation of miRNA transcription | 0.009914 | 0.039236 |
| Biological process | GO:0002763 | positive regulation of myeloid leukocyte differentiation | 0.010051 | 0.039236 |
| Biological process | GO:0071277 | cellular response to calcium ion | 0.010737 | 0.039236 |
| Biological process | GO:2000630 | positive regulation of miRNA metabolic process | 0.010737 | 0.039236 |
| Biological process | GO:0045670 | regulation of osteoclast differentiation | 0.011696 | 0.039236 |
| Biological process | GO:1902893 | regulation of miRNA transcription | 0.012107 | 0.039236 |
| Biological process | GO:0061614 | miRNA transcription | 0.012381 | 0.039236 |
| Biological process | GO:0035914 | skeletal muscle cell differentiation | 0.012929 | 0.039236 |
| Biological process | GO:0060395 | SMAD protein signal transduction | 0.014023 | 0.039236 |
| Biological process | GO:2000628 | regulation of miRNA metabolic process | 0.014023 | 0.039236 |
| Biological process | GO:0045639 | positive regulation of myeloid cell differentiation | 0.015526 | 0.039236 |
| Biological process | GO:0010586 | miRNA metabolic process | 0.016073 | 0.039236 |
| Biological process | GO:0030316 | osteoclast differentiation | 0.016892 | 0.039236 |
| Biological process | GO:0051592 | response to calcium ion | 0.017847 | 0.039236 |
| Biological process | GO:1901216 | positive regulation of neuron death | 0.018256 | 0.039236 |
| Biological process | GO:0002761 | regulation of myeloid leukocyte differentiation | 0.019618 | 0.039236 |
| Biological process | GO:0034614 | cellular response to reactive oxygen species | 0.019618 | 0.039236 |
| Biological process | GO:0071248 | cellular response to metal ion | 0.023289 | 0.04241 |
| Biological process | GO:0009612 | response to mechanical stimulus | 0.02424 | 0.042567 |
| Biological process | GO:0000302 | response to reactive oxygen species | 0.026002 | 0.043944 |
| Biological process | GO:0007179 | transforming growth factor beta receptor signaling pathway | 0.027627 | 0.043944 |
| Biological process | GO:0007519 | skeletal muscle tissue development | 0.028304 | 0.043944 |
| Biological process | GO:1902107 | positive regulation of leukocyte differentiation | 0.02871 | 0.043944 |
| Biological process | GO:1903708 | positive regulation of hemopoiesis | 0.02871 | 0.043944 |
| Biological process | GO:0060538 | skeletal muscle organ development | 0.029926 | 0.043944 |
| Biological process | GO:0045637 | regulation of myeloid cell differentiation | 0.030871 | 0.043944 |
| Biological process | GO:0071241 | cellular response to inorganic substance | 0.032895 | 0.043944 |
| Biological process | GO:0071560 | cellular response to transforming growth factor beta stimulus | 0.03303 | 0.043944 |
| Biological process | GO:0031668 | cellular response to extracellular stimulus | 0.033434 | 0.043944 |
| Biological process | GO:0002573 | myeloid leukocyte differentiation | 0.033569 | 0.043944 |
| Biological process | GO:0071559 | response to transforming growth factor beta | 0.033569 | 0.043944 |
| Biological process | GO:0034599 | cellular response to oxidative stress | 0.037201 | 0.04699 |
| Cellular component | GO:0032993 | protein-DNA complex | 0.027215 | 0.047728 |
| Cellular component | GO:0090575 | RNA polymerase II transcription regulator complex | 0.033187 | 0.047728 |
| Biological process | GO:0010038 | response to metal ion | 0.040287 | 0.050011 |
| Biological process | GO:0071496 | cellular response to external stimulus | 0.041894 | 0.050273 |
| Biological process | GO:0009410 | response to xenobiotic stimulus | 0.043098 | 0.05087 |
| Molecular function | GO:0061629 | RNA polymerase II-specific DNA-binding transcription factor binding | 0.051911 | 0.051911 |
| Biological process | GO:0062197 | cellular response to chemical stress | 0.04617 | 0.053616 |
| Biological process | GO:1902105 | regulation of leukocyte differentiation | 0.04737 | 0.054137 |
| Biological process | GO:0007517 | muscle organ development | 0.050298 | 0.056586 |
| Biological process | GO:1901214 | regulation of neuron death | 0.054016 | 0.059186 |
| Biological process | GO:0006979 | response to oxidative stress | 0.055076 | 0.059186 |
| Biological process | GO:0007178 | transmembrane receptor protein serine/threonine kinase signaling pathway | 0.055076 | 0.059186 |
| Biological process | GO:0009991 | response to extracellular stimulus | 0.056797 | 0.060138 |
| Biological process | GO:1903706 | regulation of hemopoiesis | 0.058779 | 0.061335 |
| Biological process | GO:0030099 | myeloid cell differentiation | 0.060099 | 0.061615 |
| Biological process | GO:0070997 | neuron death | 0.060759 | 0.061615 |
| Biological process | GO:0060537 | muscle tissue development | 0.066547 | 0.066547 |

# References

[1] C.R. Jack, Jr., D.A. Bennett, K. Blennow, M.C. Carrillo, B. Dunn, S.B. Haeberlein, D.M. Holtzman, W. Jagust, F. Jessen, J. Karlawish, E. Liu, J.L. Molinuevo, T. Montine, C. Phelps, K.P. Rankin, C.C. Rowe, P. Scheltens, E. Siemers, H.M. Snyder, R. Sperling, NIA-AA Research Framework: Toward a biological definition of Alzheimer's disease, Alzheimer's & dementia : the journal of the Alzheimer's Association, 14 (2018) 535-562.

[2] C.R. Jack, Jr., J.S. Andrews, T.G. Beach, T. Buracchio, B. Dunn, A. Graf, O. Hansson, C. Ho, W. Jagust, E. McDade, J.L. Molinuevo, O.C. Okonkwo, L. Pani, M.S. Rafii, P. Scheltens, E. Siemers, H.M. Snyder, R. Sperling, C.E. Teunissen, M.C. Carrillo, Revised criteria for diagnosis and staging of Alzheimer's disease: Alzheimer's Association Workgroup, Alzheimer's & dementia : the journal of the Alzheimer's Association, DOI 10.1002/alz.13859(2024).

[3] D.E. Battle, Diagnostic and Statistical Manual of Mental Disorders (DSM), CoDAS, 25 (2013) 191-192.

[4] H. Yu, S. Sun, J. Ling, H. Chen, G. Liu, Influence of health literacy on health outcomes of different social strata-- an empirical study based on the data of China's health literacy investigation, International journal for equity in health, 22 (2023) 42.

[5] J. Ignacio de Ulíbarri, A. González-Madroño, N.G. de Villar, P. González, B. González, A. Mancha, F. Rodríguez, G. Fernández, CONUT: a tool for controlling nutritional status. First validation in a hospital population, Nutricion hospitalaria, 20 (2005) 38-45.

[6] G.P. Buzby, J.L. Mullen, D.C. Matthews, C.L. Hobbs, E.F. Rosato, Prognostic nutritional index in gastrointestinal surgery, American journal of surgery, 139 (1980) 160-167.

[7] O. Bouillanne, G. Morineau, C. Dupont, I. Coulombel, J.P. Vincent, I. Nicolis, S. Benazeth, L. Cynober, C. Aussel, Geriatric Nutritional Risk Index: a new index for evaluating at-risk elderly medical patients, The American journal of clinical nutrition, 82 (2005) 777-783.

[8] V.P. Leung, L.C. Lam, H.F. Chiu, J.L. Cummings, Q.L. Chen, Validation study of the Chinese version of the neuropsychiatric inventory (CNPI), International journal of geriatric psychiatry, 16 (2001) 789-793.

[9] M. Hamilton, A rating scale for depression, Journal of neurology, neurosurgery, and psychiatry, 23 (1960) 56-62.

[10] M. Hamilton, The assessment of anxiety states by rating, The British journal of medical psychology, 32 (1959) 50-55.

[11] R. Katzman, M.Y. Zhang, Q. Ouang Ya, Z.Y. Wang, W.T. Liu, E. Yu, S.C. Wong, D.P. Salmon, I. Grant, A Chinese version of the Mini-Mental State Examination; impact of illiteracy in a Shanghai dementia survey, Journal of clinical epidemiology, 41 (1988) 971-978.

[12] J. Yu, J. Li, X. Huang, The Beijing version of the Montreal Cognitive Assessment as a brief screening tool for mild cognitive impairment: a community-based study, BMC psychiatry, 12 (2012) 156.

[13] M. Marvardi, P. Mattioli, L. Spazzafumo, R. Mastriforti, P. Rinaldi, M.C. Polidori, A. Cherubini, R. Quartesan, L. Bartorelli, S. Bonaiuto, D. Cucinotta, A. Di Iorio, M. Gallucci, M. Giordano, M. Martorelli, G. Masaraki, A. Nieddu, C. Pettenati, P. Putzu, V. Solfrizzi, A.E. Tammaro, P.F. Tomassini, C. Vergani, U. Senin, P. Mecocci, The Caregiver Burden Inventory in evaluating the burden of caregivers of elderly demented patients: results from a multicenter study, Aging clinical and experimental research, 17 (2005) 46-53.

[14] B. Vellas, Y. Guigoz, P.J. Garry, F. Nourhashemi, D. Bennahum, S. Lauque, J.L. Albarede, The Mini Nutritional Assessment (MNA) and its use in grading the nutritional state of elderly patients, Nutrition (Burbank, Los Angeles County, Calif.), 15 (1999) 116-122.

[15] X.M. Wang, W.F. Zhong, Z.H. Li, P.L. Chen, Y.J. Zhang, J.J. Ren, D. Liu, Q.Q. Shen, P. Yang, W.Q. Song, F. Liang, Y. Nan, J.X. Xiang, Y.R. Wu, Y.B. Lv, X. Gao, V.B. Kraus, X.M. Shi, C. Mao, Dietary diversity and frailty among older Chinese people: evidence from the Chinese Longitudinal Healthy Longevity Study, The American journal of clinical nutrition, 117 (2023) 383-391.

[16] D. Liu, W.T. Zhang, J.H. Wang, D. Shen, P.D. Zhang, Z.H. Li, P.L. Chen, X.R. Zhang, Q.M. Huang, W.F. Zhong, X.M. Shi, C. Mao, Association between Dietary Diversity Changes and Cognitive Impairment among Older People: Findings from a Nationwide Cohort Study, Nutrients, 14 (2022).

[17] N.S. Pentkowski, K.K. Rogge-Obando, T.N. Donaldson, S.J. Bouquin, B.J. Clark, Anxiety and Alzheimer's disease: Behavioral analysis and neural basis in rodent models of Alzheimer's-related neuropathology, Neuroscience and biobehavioral reviews, 127 (2021) 647-658.

[18] R. Dang, M. Wang, X. Li, H. Wang, L. Liu, Q. Wu, J. Zhao, P. Ji, L. Zhong, J. Licinio, P. Xie, Edaravone ameliorates depressive and anxiety-like behaviors via Sirt1/Nrf2/HO-1/Gpx4 pathway, Journal of neuroinflammation, 19 (2022) 41.

[19] H. Han, M. Xu, J. Wang, M.D. Li, Z. Yang, CRISPR/Cas9 based gene editing of Frizzled class receptor 6 (FZD6) reveals its role in depressive symptoms through disrupting Wnt/β-catenin signaling pathway, Journal of advanced research, 58 (2024) 129-138.

[20] A. Vilella, M. Bodria, B. Papotti, I. Zanotti, F. Zimetti, G. Remaggi, L. Elviri, F. Potì, N. Ferri, M.G. Lupo, G. Panighel, E. Daini, E. Vandini, M. Zoli, D. Giuliani, F. Bernini, PCSK9 ablation attenuates Aβ pathology, neuroinflammation and cognitive dysfunctions in 5XFAD mice, Brain, behavior, and immunity, 115 (2024) 517-534.
